# Ultramafic specialist lichens persist through flexible symbioses structured by climate-driven filtering and modulated by substrate effects

**DOI:** 10.3897/imafungus.17.199447

**Published:** 2026-08-24

**Authors:** Miroslav Caboň, Marek Slovák, Marek Svitok, Jana Steinová, Zuzana Fačkovcová, Jaroslava Gužiková, Jaromír Kučera, Luca Paoli, Tomáš Větrovský, Alica Hindáková, Dušan Senko, Judita Zozomová-Lihová, Andrea Melichárková, Zuzana Gajdošová, Anna Guttová

**Affiliations:** 1 Plant Science and Biodiversity Centre, Slovak Academy of Sciences, Bratislava, Slovakia Department of Biology and General Ecology, Faculty of Ecology and Environmental Sciences, Technical University in Zvolen Zvolen Slovakia; 2 Department of Botany, Faculty of Science, Charles University, Prague, Czech Republic Department of Botany, Faculty of Science, Charles University Prague Czech Republic; 3 Department of Biology and General Ecology, Faculty of Ecology and Environmental Sciences, Technical University in Zvolen, Zvolen, Slovakia Institute of Microbiology, Academy of Sciences of the Czech Republic Prague Czech Republic; 4 Department of Forest Ecology, Faculty of Forestry and Wood Sciences, Czech University of Life Sciences Prague, Prague, Czech Republic Department of Biology, University of Pisa Pisa Italy; 5 Jessenius Faculty of Medicine in Martin, Comenius University Bratislava, Martin, Slovakia Plant Science and Biodiversity Centre, Slovak Academy of Sciences Bratislava Slovakia; 6 Department of Biology, University of Pisa, Pisa, Italy Department of Forest Ecology, Faculty of Forestry and Wood Sciences, Czech University of Life Sciences Prague Prague Czech Republic; 7 Institute of Microbiology, Academy of Sciences of the Czech Republic, Prague, Czech Republic Jessenius Faculty of Medicine in Martin, Comenius University Bratislava Martin Slovakia

**Keywords:** Endolichenic fungi, holobiont, lichen, metabarcoding, *
Symbiochloris
*, ultramafites

## Abstract

Ultramafic substrates impose severe edaphic stress characterised by metal toxicity, nutrient imbalance, and extreme microclimatic conditions, creating spatially fragmented habitats that can shape evolutionary and ecological dynamics of associated organisms. Together with climatic variation, such factors may also influence the structure of holobiont communities of lichenized fungi. Here we investigated the ultramafic specialist lichen *Solenopsora liparina* across its entire range. We contextualized its symbiotic associations using calcicolous congeners (*S. candicans* and *S. cesatii*), as ecological reference taxa representing contrasting substrate-associated lineages within the sampled dataset, to assess how climatic filtering acting within a specialised edaphic niche structures symbiotic partner diversity and composition. Photobionts and endolichenic fungi displayed climate-associated compositional turnover and lineage-level diversity. Variation in the composition of these symbionts was most strongly associated with precipitation during the warmest quarter and, in photobionts, with altitude and temperature seasonality. In photobionts, dominant lineages differed in their relative occurrence among host taxa, whereas low-abundance lineages were broadly shared across samples within the dataset. Despite this turnover, alpha diversity remained generally stable across environmental gradients, indicating that climatic variation most strongly affects community composition rather than within-sample diversity. The results are consistent with a model of mixed host- and substrate- associated symbiont assembly, in which dominant photobiont lineages exhibit host- or substrate-linked preferences, whereas low-abundance associates show weaker specificity and broader ecological overlap across environmental and host contexts. Overall, the findings indicate that climatic factors structure the composition of symbiotic partners without strongly altering their overall diversity, while variation among host taxa is reflected in lineage turnover of dominant symbionts. The persistence of the ultramafic specialist lichen *S. liparina* thus appears to rely on flexible, compositionally dynamic symbiotic associations shaped by climatic variation within a spatially and edaphically constrained niche, rather than obligate partner specificity. Substrate effects are interpreted here as host-associated lineage patterns rather than independently quantified drivers. More broadly, the results suggest that climatic variation can in some cases strongly influence symbiotic assembly within environmentally extreme and spatially heterogeneous systems. Substrate specialisation provides the ecological context in which such interactions occur. Together, the findings highlight the importance of climatic variation in shaping symbiotic assembly within an edaphically specialised system, emphasizing that patterns associated with host and substrate context are expressed mainly through lineage turnover rather than wholesale community replacement.

## Introduction

Ultramafic rocks, often exposed within ophiolite complexes originating from obducted oceanic lithosphere, occur as outcrops or sparsely vegetated landforms (Anacker 2014) and represent sharply delineated, low-competition edaphic islands (Rajakaruna 2017). These habitats present highly stressful conditions due to distinct bioavailable elemental profiles (e.g., toxic levels of Mg, Ni, Cr, Co; limited macronutrients such as N, P, K, and Ca), along with low water-holding capacity, high UV radiation, and heat retention from dark-coloured bedrock. Such factors pose substantial adaptive challenges, often leading to ecological divergence and local adaptation, particularly well documented in plants, where ultramafic substrates have been linked to population differentiation and speciation processes (Anacker et al. 2010; Armbruster 2014; Rajakaruna et al. 2014; Arnold et al. 2016; Echevarria 2018; Sianta and Kay 2019; Konečná et al. 2020; Kierczak et al. 2021). In bacteria and other microbial communities, studies have primarily reported shifts in community composition and metal tolerance rather than clear cases of ecological speciation (e.g. Kumar et al. 2023). Several plant families have become model systems for studying adaptation, constitutive tolerance, evolution, biodiversity, and ecology (Cacho and Strauss 2014; Rajakaruna et al. 2014; Sianta and Kay 2019; Konečná et al. 2020, 2022). Organisms thriving on ultramafic substrates employ diverse adaptive strategies, such as hyperaccumulation or exclusion of toxic elements (Favero-Longo et al. 2004; Vithanage et al. 2019). Physiological adaptation is often supported by cross-kingdom interactions, including mycorrhizal associations (Kazakou et al. 2008; Doubková et al. 2012; Jourand et al. 2014) or rhizobacterial symbioses (Sujkowska-Rybkowska et al. 2020). While the ecological importance of ectomycorrhizal fungi has been highlighted (Branco and Ree 2010), their diversity may be constrained by high metal concentrations and low Ca/Mg ratios, which inhibit fungal growth (Kazakou et al. 2008; Rajakaruna et al. 2009; Branco et al. 2022).

Focusing on symbiotic interactions, lichens represent an intricate system in which fungal partners (mycobionts) associate with photoautotrophic organisms (photobionts) and both are further coupled with diverse microbial communities such as endolichenic yeast-like fungi and dark-septate endophytes (Spribille 2018; Keller et al. 2024). Lichen symbioses occurring on extreme edaphic substrates, including metal-rich systems like ultramafites, involve mycobiont–photobiont associations in which photobiont performance may vary under elevated metal concentrations, as demonstrated by experimental work showing that copper tolerance can arise through selection for metal-tolerant photobiont strains (Bačkor and Váczi 2002). However, no consistent relationship between substrate chemistry and photobiont diversity at higher taxonomic levels has been observed (Bačkor and Fahselt 2008; Bačkor et al. 2010) and its role in structuring endolichenic fungal communities also remains largely unexplored (Suryanarayanan and Thirunavukkarasu 2017). This raises a question: how do such toxic environments influence the diversity of complex symbiotic systems – lichen holobionts integrating mycobionts, photobionts, and associated microbiota (Tripp et al. 2017; Spribille 2018; Mulroy et al. 2022; Vondrák et al. 2023)? The composition and diversity of these partners likely play a fundamental role in shaping symbiotic performance, particularly in enhancing edaphic tolerance and nutrient acquisition, and thus contribute to the adaptation and evolution of these complex biological associations (Branco and Ree 2010; Doubková et al. 2012). Indeed, lichen mycobionts can associate with various locally available photobionts, which may play a dominant role in structuring symbiotic partnerships (Leavitt et al. 2015). Variation in photobiont identity across environmental gradients (Medeiros et al. 2021; Vančurová et al. 2021; Stanton et al. 2022; Rolshausen et al. 2023) suggests that photobiont turnover supports acclimation to extreme conditions. Although lichen-forming fungi are generally thought to associate with photobionts available in the local environment (Steinová et al. 2022), again it remains unclear whether this pattern holds in ultramafic habitats, where strong edaphic filtering, patchy host distribution, and spatial habitat discontinuity could potentially limit photobiont availability and lead to substrate-associated structuring. To date, published studies have mainly documented the occurrence and level of specialisation of lichen mycobionts to ultramafic substrates (Favero-Longo et al. 2018; Mulroy et al. 2022). Only eight lichen species are known exclusively from ultramafic substrates (Medeiros et al. 2014; Favero-Longo et al. 2018; Mulroy et al. 2022), yet their role as hosts of potentially distinct symbiotic communities remains essentially unknown (Rajakaruna et al. 2014).

Apart from edaphic conditions, climatic factors such as temperature and precipitation may also influence the symbiotic partners in lichen thalli by shaping photobiont composition and availability, with consequences for community assembly, ecological interactions, and potentially long-term evolutionary dynamics (e.g. Peksa and Škaloud 2011; Rodríguez‐Arribas et al. 2023). Environmental stressors, such as extreme light and climatic conditions, can reduce photobiont fitness, constrain their distribution, and shape lichen symbiotic communities (Beckett et al. 2021; Peksa et al. 2022). So it is reasonable to expect that climate also plays a vital role in shaping the diversity, distribution, and survival of ultramafic holobionts. Revealing how climate interacts with edaphic factors is essential not only for understanding the diversity and evolution of ultramafic-associated lichens but also for predicting their response to future environmental changes (Rajakaruna 2017; Favero-Longo et al. 2018).

Studies of lichens in harsh environments such as seasonally arid montane habitats (Harutyunyan et al. 2008), subalpine rocky zones (Fernández-Mendoza et al. 2017), alpine rock habitats (Muggia et al. 2016), or the arid, cold conditions of Antarctica (Selbmann et al. 2013) indicate that endolichenic fungi, including black fungi may play important roles in stress tolerance and functioning of complex symbiotic system (Muggia and Grube 2018). In plant systems, ultramafic habitats are often associated with strong environmental filtering and spatial structuring of plant communities (Rajakaruna et al. 2014); however, whether similar processes shape lichen symbioses remains unknown. We therefore investigate whether ultramafic substrates influence the diversity and community composition of lichen symbionts, and whether such habitats promote patterns of ecological filtering or diversification. Although empirical data are still limited, studies in plants and mycorrhizal fungi suggest that ultramafic substrates can promote both ecological filtering and evolutionary diversification (e.g., Anacker 2014; Crossay et al. 2018), making similar patterns plausible in lichen symbioses.

**Figure 1.**
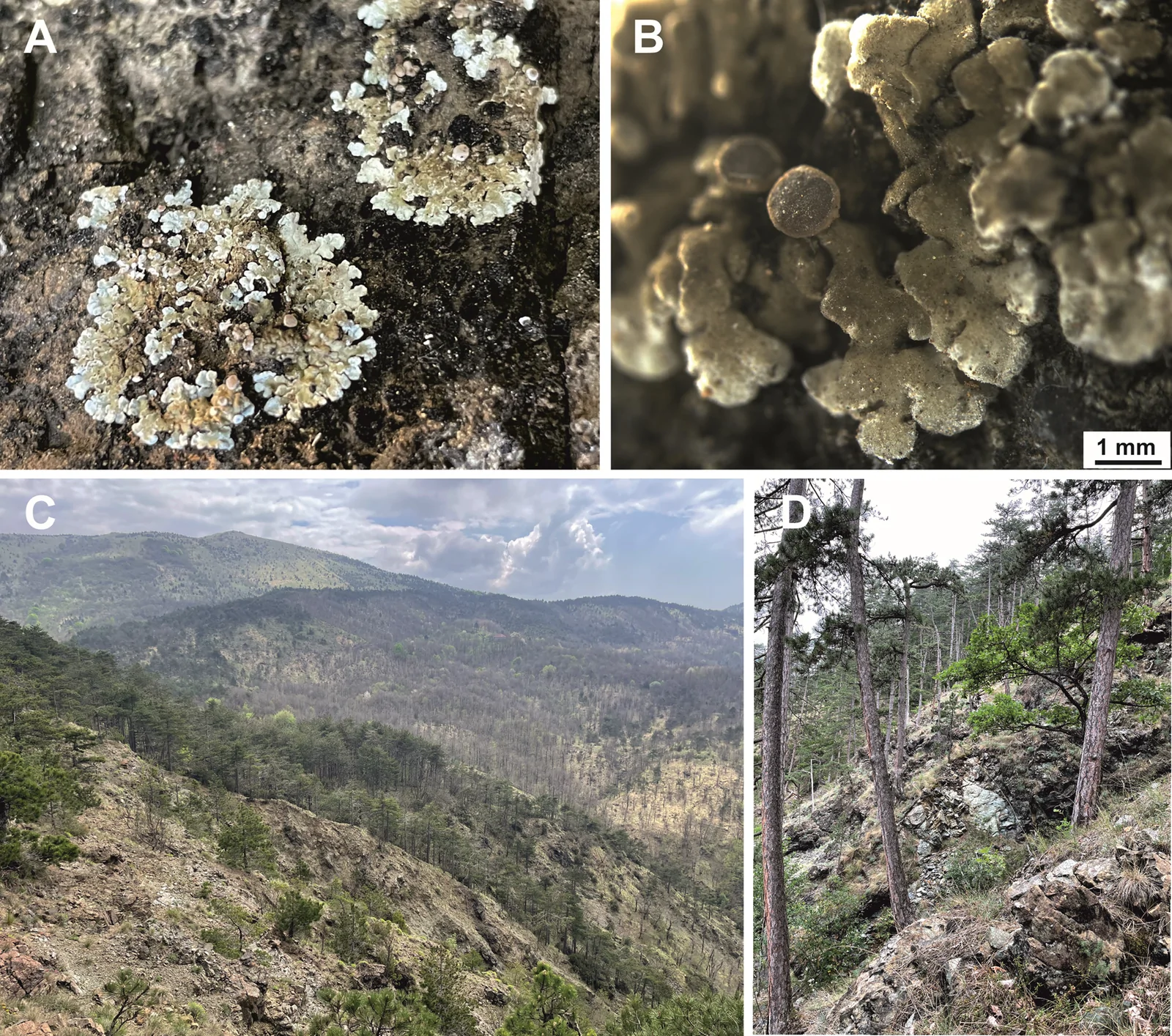
**A** Ultramafic specialist *Solenopsora liparina* – placodioid thalli; **B***S. liparina* – fruiting bodies (apothecia) and lobes with pruinose tips; **C, D** natural habitats with ultramafic outcrops in Italy, Liguria (**C**) and Bosnia and Herzegovina, Smolin Mts (**D**).

The focal species introduced in this study – the saxicolous lichen *Solenopsora liparina* (Nyl.) Zahlbr. (*Catillariaceae*), occurs on ultramafic rocks in the Mediterranean Basin and Central and Western Europe (Fig. 1). These ultramafic complexes, primarily of Jurassic–Cretaceous and Devonian-Permian origin, form a discontinuous belt extending from Great Britain through the Massif Central, the Hercynian Mountains, the Apennines, and the Balkans to Western Asia (Vaughan and Scarrow 2003; Dilek and Furnes 2009; Guttová et al. 2019). The distribution of *S. liparina* is predominantly associated with the Mediterranean climate zone, while also extending into Atlantic and continental climate regions (Guttová et al. 2019). The species does not form asexual propagules (Guttová et al. 2014) which would allow joint dispersal of all symbiotic partners. Instead, the mycobiont produces sexual spores, which must subsequently associate with compatible photobionts and additional symbionts to initiate thallus formation.

Ultramafic ecosystems represent natural laboratories for studying how specific edaphic conditions and climate jointly influence holobiont assembly, yet their role in shaping lichen symbioses across full species’ distributional ranges remains poorly resolved. In this work, we investigate whether and how edaphic and climatic factors shape the distribution of the lichen *S. liparina*, as well as the diversity and community composition of its symbiont partners. Using a comprehensive dataset spanning the full distribution range of the species, we integrate ecological niche modeling within a biogeographical framework to identify climatic drivers of habitat suitability and range limits. This analysis is applied in the context of ultramafic specialisation, with calcicolous congeners included as ecological reference taxa representing a natural ecological contrast in substrate-associated conditions ranging from ultramafic to calcareous environments. In parallel, metabarcoding data are used to assess how climatic variability and the spatial discontinuity of ultramafic habitats are associated with the variation in photobiont and endolichenic fungal communities. Overall, these approaches provide a multi-symbiont perspective on how environmental gradients and substrate context relate to patterns of symbiotic assembly across scales. We hypothesize that symbiont diversity and community composition are associated with climatic conditions and substrate-related ecological context, with substrate effects expressed through lineage-level associations among host systems. We further ask whether climatic conditions and substrate-associated host context are reflected in consistent but potentially overlapping patterns of symbiont turnover across analytical frameworks.

## Materials and methods

### Analytical workflow

Our approach (Suppl. material 1: fig. S1) combines ecological and genetic data, linking respective analyses. Pre-processed and quality-controlled ecological data related to the focal species *S. liparina* and two sister congeners, *S. candicans* (Dicks.) Steiner and *S. cesatii* (A. Massal.) Zahlbr., confined to calcareous rocks, were incorporated into climatic niche models, including assessments of climatic suitability and potential distribution patterns, to test whether climatic conditions shape the composition of lichen-associated assemblages. We established a phylogenetic framework for the mycobiont of *S. liparina* by generating multilocus Sanger sequence data to infer the evolutionary relationships relative to its calcicolous congeners. The dataset included newly generated multilocus sequences of *S. liparina*, while sequences of calcicolous congeners (cf. Guttová et al. 2014; Fačkovcová et al. 2020) were retrieved from GenBank. Molecular data (Sanger sequences and metagenomic data) related to the photobionts and endolichenic fungi associated with *S. liparina* were generated and analysed to establish taxonomic identities, reconstruct phylogenetic relationships (of photobionts), identify functional groups, and explore diversity, community composition, and indicator lineages in relation to climatic context. The outputs enable an ecological inference at both the holobiont level and the level of individual components of the lichen.

### Ecological niches and climatic drivers of lichen symbiosis

To provide insight into the relative importance of bioclimatic variables contributing most significantly to the distribution of the ultramafic specialist *S. liparina*, habitat suitability models (HSMs) were constructed following Senko et al. (2024), based on 24 confirmed occurrences derived from field observations and revised material from 20^th^ century (Suppl. material 3: table S1). Its climatic requirements were studied in the context of two sister congeners confined to calcareous sedimentary rocks – *S. candicans*, and *S. cesatii*, which show strongly conserved habitat and climate affinities across their ranges (Fačkovcová et al. 2017). HSMs for these species were based on 298 (*S. candicans*) and 117 (*S. cesatii*) localities, respectively. Models were implemented in MaxEnt v3.4.4 (Steven et al. 2020) using the cloglog output format with 10 replicate runs. For taxa with more than 100 occurrence records, cross-validation was used as a replication approach, whereas bootstrap resampling was applied for *S. liparina* with fewer occurrences. Model performance was evaluated by area under curve metric (AUC; Fielding and Bell 1997).

We compared the environmental niches of the three lichen species using the kernel‐smoothed framework (Broennimann et al. 2012). A computational region corresponding to the distribution range of the three congeners was first defined, with a spatial resolution set to 0°08'. Within this framework, a regular grid of points covering the entire distribution areas of the three congeners was generated. A uniform raster with all cells assigned a value of 1 was created and subsequently converted to a point vector dataset, resulting in a regular network of points across the species’ range. For each point, values of the 19 bioclimatic variables (Suppl. material 3: table S2) were extracted from the corresponding raster layers. All spatial analyses were carried out in the GRASS GIS v7 environment (GRASS development team 2016) in Computing Center of the Slovak Academy of Sciences, Bratislava (Devana supercomputer). Bioclimatic variables were standardized and summarized by principal component analysis (PCA) on the correlation matrix. The first two principal components defined a 300 × 300 grid, each cell representing a unique set of environmental conditions. Lichen species occurrences were binned to cells and converted to densities, which were then smoothed with a Gaussian kernel to reduce the influence of uneven sampling intensity and spatial recording bias for niche overlap (Broennimann et al. 2012; Guisan et al. 2012). Smoothed occurrence densities were visualized in PCA space, and pairwise niche overlap was quantified with Schoener’s D (0 = no overlap, 1 = complete overlap; Schoener 1968). The significance of observed D values was evaluated using niche equivalency and niche similarity tests (Warren et al. 2008). The equivalency test assessed whether two niches were identical by randomizing species identities within pooled occurrences (1,000 permutations) while preserving sample sizes. We rejected the null when observed D fell below the 5^th^ percentile of the null distribution (one‐tailed test). We also conducted the similarity test, which asks whether niches are more similar than expected by chance given the available environmental space. We generated a null distribution by random reallocations within the background and rejected the null when observed D exceeded the 95^th^ percentile (one‐tailed test).

### Sampling design for genetic data

**Figure 2.**
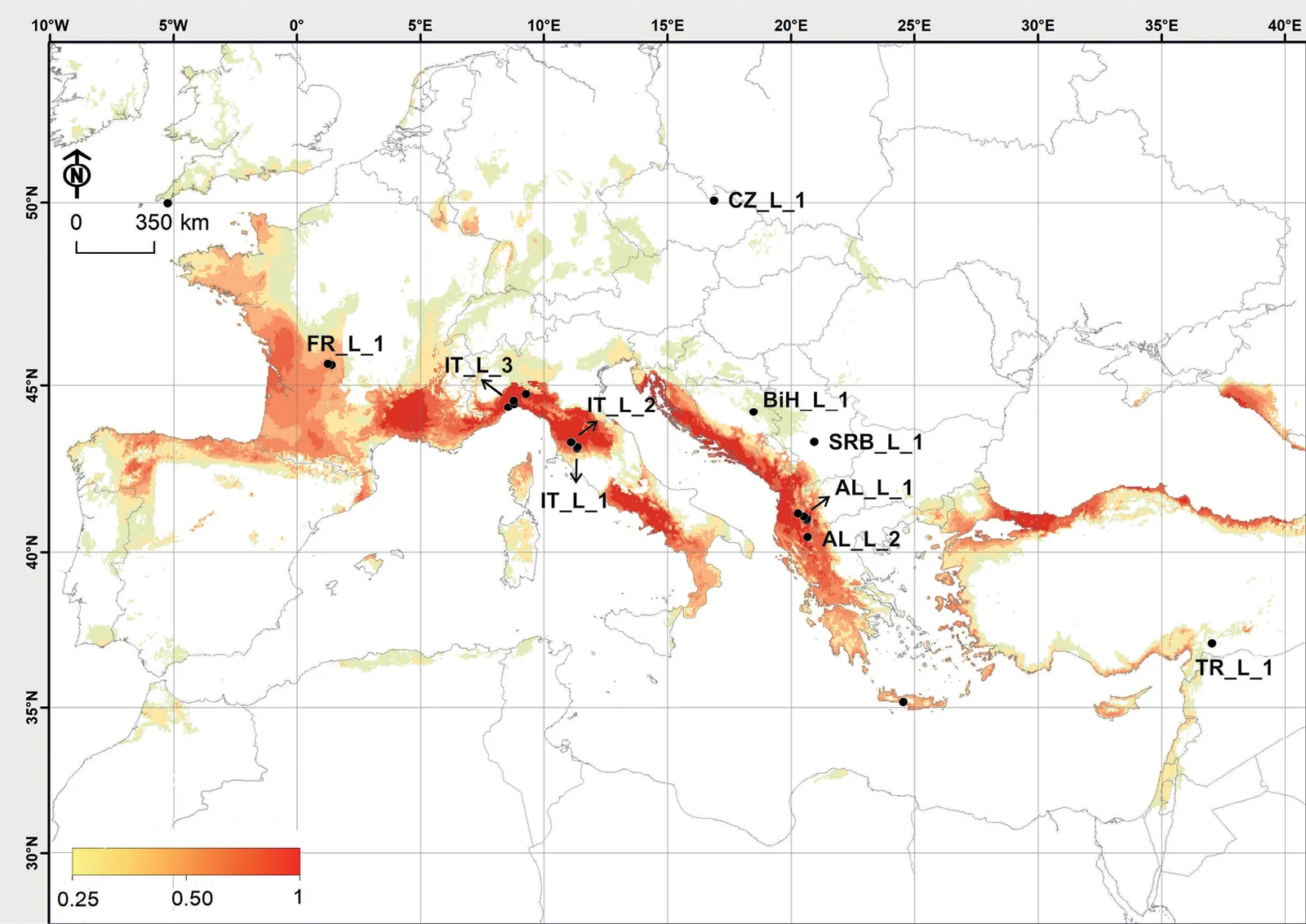
Projection of recent HSM of *Solenopsora liparina* across its range. Colours indicate the predicted habitat suitability ranging from 0.25 (low suitability) to 1.0 (high suitability). Black dots represent known occurrence records of *S. liparina*. Localities from which samples were collected for the molecular analysis are labeled with their population abbreviations (see table S3). Light green shading indicates predicted habitat suitability values < 0.25, which fall below the range represented by the colour scale in the figure legend.

In this study, we included samples from the vast majority of known *S. liparina* localities. In total, we investigated 32 specimens, originating from 10 populations (2–5 specimens per population; Fig. 2, Suppl. material 3: table S3). The relatively low number of specimens per population reflects its rarity in the given sampling sites. A set of samples (10) of the two congeners, which are restricted to calcareous substrates, was included to gain insight into potential differences among the microbiomes of ultramafic and limestone taxa and exploratory comparison (Suppl. material 3: table S3).

### DNA extraction

Lichen thalli were physically cleaned of debris and washed with 0.5% NaOCl to sterilize their surfaces and avoid contamination by extrathalline microorganisms (Muggia and Grube 2018). Total genomic DNA was extracted from the marginal parts of thalline lobes by a DNeasy® Plant Mini Kit (Qiagen, Hilden, Germany) following manufacturer's recommendations. Quality and concentration of DNA were checked by a Qubit 4.0 fluorimeter (ThermoFischer Scientific) and BioSpec-nano spectrophotometer (Shimadzu Biotech). The DNA extracts were used to produce both Sanger sequences as well as short metabarcodes. Sanger sequencing provided high-confidence identification of dominant symbiotic partners for phylogenetic inference, whereas metabarcoding captured the broader holobiont diversity, including low-abundance taxa.

### Sanger sequencing

Prior to the profiling of the intrathalline microbiome, the genetic diversity of mycobiont and dominant photobionts was inspected using Sanger sequencing. For this purpose, the internal transcribed spacer (ITS) region (ITS1-5.8S-ITS2) of the nuclear rDNA, the ketosynthase (KS) domain of fungal type I polyketide synthase genes (PKS), and a protein-coding nuclear gene *MCM7* (replication licencing factor) were amplified using fungal specific primers (Suppl. material 3: table S4). The protein coding chloroplast *rbcL* gene and nuclear ITS region of predominant photobionts were amplified employing algal specific primers (Suppl. material 3: table S4). Amplification of the KS domain and *MCM7* gene was performed on a limited number of samples, as the selected dataset based on preliminary results of variability inferred from the ITS dataset and also spanning the species’ distribution area showed minimal genetic variability. Additionally, sequences for the dominant photobiont could not be obtained for some samples, despite multiple amplification attempts. The sequences are deposited in the GenBank database under accession numbers listed in Suppl. material 4: table S15.

### Metabarcoding

The DNA extracts were used for ITS2 rRNA gene amplicon sequencing. Two consecutive PCR reactions per sample were used, using normal and barcode fusion primers. Primers ITS3 and ITS4 (White et al. 1990) were used for amplification of the target marker. The quality of the library product was checked using gel electrophoresis. PCR products were purified with the Agencourt Ampure XP system (Beckman Coulter, Brea, CA, USA), after which a Qubit 2.0 fluorimeter (Life Technologies, MA, USA) was used to measure the concentration of the purified PCR products. Then, barcode-tagged amplicons from the different samples were mixed in equimolar concentrations. Templating and enrichment for sequencing were performed using the One-Touch 2 and One-Touch ES systems (Life Technologies, USA), with the sequencing of the amplicons performed on the Ion Torrent Personal Genome Machine (PGM) using the Ion PGM Hi-Q Sequencing Kit with the Ion 314 Chip v. 2 (Thermo Fisher Scientific®, USA), following the manufacturer‘s instructions.

### Phylogenetic inference of the mycobiont

To assess the phylogenetic position of the mycobiont *S. liparina*, a concatenated dataset comprising ITS, the KS domain of PKS, and *MCM7* sequences was analysed. The dataset included newly generated Sanger sequences, which were incorporated into the alignment framework established in a previous phylogenetic study of the genus *Solenopsora* (Guttová et al. 2014). All mycobiont sequences were edited and aligned in Geneious Prime v2025.2.1 (Biomatters Ltd., Auckland, New Zealand) using the built-in alignment algorithm, followed by manual inspection and adjustment. The alignment used in the analyses is deposited in Figshare at doi: 10.6084/m9.figshare.31081510. The exact boundaries of the 5.8S rRNA gene within the ITS region (i.e., between ITS1 and ITS2) were determined using ITSx (Bengtsson-Palme et al. 2013). The optimal partitioning scheme and substitution models for phylogenetic analyses were selected using PartitionFinder v2.1.1 (Lanfear et al. 2016) under the greedy search algorithm. The best-fit scheme partitioned the alignment into seven subsets: 1 – (GTR+I+G) ITS1 and ITS2, 2 – (K80+I) 5.8S rRNA gene, 3 – (GTR+I+G) first codon positions of *MCM7* and PKS, 4 – (HKY+I) second codon positions of *MCM7* and PKS, 5 –(HKY+G) third codon position of *MCM7*, 6 – (HKY) third codon position of PKS, and 7 – (K80+I) intron region of PKS. Phylogenetic relationships were inferred using both Bayesian inference (BI) and maximum likelihood (ML) approaches. Bayesian analyses were conducted in MrBayes v3.2.7a (Ronquist and Huelsenbeck 2003) using a Markov Chain Monte Carlo (MCMC) approach with four chains run for 10 million generations and sampled every 1,000 generations. Convergence was assessed based on standard diagnostics, and the first 25% of trees were discarded as burn-in prior to constructing a 50% majority-rule consensus tree. Maximum Likelihood analyses were performed using RAxML v8.2.11 (Stamatakis 2014) under the GTRGAMMA substitution model. Branch support was evaluated using 1,000 bootstrap replicates.

### Metabarcoding data processing

ITS2 data were used to investigate the lichen microbiome, focusing on endolichenic fungi, predominant and additional photobionts, as well as potential ecological associations with microbiome diversity. Amplicon sequence data were processed using the SEED 2.0.1. pipeline (Větrovský et al. 2018). The sequencing outputs were quality-checked, and all sequences with more than 2 mismatches in the primer region, any mismatch in the tag region, or an overall quality Phred score (qm) lower than 25 were discarded. The whole ITS2 region was retrieved using ITSx v. 1.0.11 (Bengtsson-Palme et al. 2013). For taxonomic analysis, we carried out independent clustering of sequences into Operational Taxonomic Units (OTUs) using UPARSE in USEARCH v 11.0.667 (Edgar 2013), applying a 97% similarity threshold. De novo detected chimeric sequences and low-abundance OTUs (those with fewer than 5 sequences) were discarded, yielding 1,047 OTUs (comprising 1,600,562 reads). The most abundant sequence from each OTU was retrieved for subsequent taxonomic and functional guild assignments. Metabarcoding data are available in the NCBI Sequence Read Archive (SRA) under submission PRJNA1392552.

### Taxonomy and functional guilds assignment

The most abundant sequence variant of each OTU was BLAST-matched against the UNITE all eukaryotes ITS reference database release 9.0 (Nilsson et al. 2019; Kõljalg et al. 2020). Initial fungal functional guild assignments to OTUs were done using the FungalTraits database (Põlme et al. 2020). Based on published ecological information and functional trait assignments, OTUs belonging to taxa reported as yeasts or yeast-like fungi were retrieved and retained for subsequent analyses as endolichenic yeasts. Identity and species delimitation of the endolichenic fungi and Viridiplantae photobionts were further refined by additional phylogenetic analyses based on expanded datasets with additional NCBI blast and retrievement of referential sequences, altogether with selection of additional referential sequences published in Gheza et al. (2025), Miral et al. (2022), Vančurová et al. (2015), Cometto et al. (2023), and Medeiros et al. (2021). Newly generated photobiont ITS2 sequences obtained by Sanger sequencing were also included in the photobiont dataset. Both datasets (endolichenic fungi and photobionts) were analysed independently and aligned by MAFFT online service (Katoh et al. 2019) using Q-INS-i setting and further manually curated in Geneious R10. Curated datasets were uploaded to the CIPRES portal (Miller et al. 2010), and Maximum Likelihood phylogenetic analyses were calculated using RAxML-HPC Blackbox under the GTR+Gamma substitution model with 1000 replications. In addition, two further photobiont alignments comprising ITS (ITS1, 5.8S, ITS2) and *rbcL* sequences were analysed using the same maximum likelihood framework, to validate taxonomic assignments inferred from ITS2 data and to assess congruence of phylogenetic placement across independent molecular markers. In the context of photobiont plurality, we follow the terminology according to Dědková et al. (2025).

### Environmental drivers of biont diversity and community composition

We quantified alpha diversity of photobionts and endolichenic fungi associated with *S. liparina* using Hill numbers (true diversities) of orders 0 (species richness), 1 (Shannon diversity, i.e., the exponential of Shannon entropy), and 2 (Simpson diversity, i.e., the inverse Simpson index), following Jost (2006). We also summarized community evenness using Hill relative evenness for orders 1 (Shannon evenness) and 2 (Simpson evenness), based on Jost (2010). Before calculating these metrics, sequence reads for each sample were normalized (rarefied) to match the abundance of the least-read sample. To ensure increased accuracy, sample rarefaction and alpha diversity measures were computed in 10 replications, each with a different set of reads in SEED 2.1.2 (Větrovský et al. 2018), and subsequently averaged. Multivariate analyses were performed on the dataset transformed to relative read abundances. Community composition was assessed as multivariate variation in species abundance data using PERMANOVA based on Bray-Curtis dissimilarities. Prior to the calculation of relative read abundance for each OTU, all mycobiont reads belonging to *Solenopsora* were discarded from the analysis, and for the endolichenic fungal dataset, photobiont reads were additionally removed prior to downstream analyses.

Finally, we investigated environmental drivers of the photobiont and endolichenic fungal variables described above using generalized linear mixed models (GLMMs; Bolker et al. 2009) and distance-based redundancy analysis (db-RDAs; Legendre and Anderson 1999). Altitude and selected bioclimatic variables were used as environmental predictors, with population included as a random intercept in GLMMs to account for the hierarchical sampling design. Integer-valued richness variables were modeled assuming Poisson errors with a logarithmic link, continuous diversity indices were modeled using Gaussian errors with an identity link, and evenness metrics bounded between 0 and 1 were analysed using beta regression with a logit link (Ferrari and Cribari-Neto 2004). Model assumptions and fit were assessed using simulation-based residual diagnostics (Dunn and Smyth 1996). The significance of fixed effects was evaluated using likelihood-ratio tests, with the test statistic referred to a χ^2^ distribution, for the Poisson models (McCullagh and Nelder 1989) and beta models (Ferrari and Cribari-Neto 2004). For Gaussian models, we employed F-tests with Satterthwaite-adjusted degrees of freedom (Kuznetsova et al. 2017). To avoid multicollinearity among predictors (Dormann et al. 2013), we assessed pairwise correlations and removed one variable from any pair with |r| > 0.7. The final set of predictors comprised altitude and temperature annual range (BIO7), precipitation of wettest month (BIO13), precipitation seasonality (BIO15), precipitation of warmest quarter (BIO18), and precipitation of coldest quarter (BIO19). We first fitted full models including all predictors and then iteratively removed variables with variance inflation factors > 5. Final models were obtained using backward elimination to retain the most parsimonious predictor set (Crawley 2012). For db-RDAs, OTU relative abundances of photobionts and endolichenic fungi were square-root transformed to reduce the influence of dominant taxa (Clarke et al. 2014), and Bray–Curtis dissimilarities (Bray and Curtis 1957) were calculated from transformed community matrices. Environmental variables were fitted as constraining predictors in db-RDAs, and significance was assessed using permutation tests with 10,000 restricted permutations that respected the hierarchical sampling structure by permuting entire populations while retaining within-population sample structure. The analyses were performed in R v. 4.2.2 (R Core Team 2022) using the libraries bestNormalize v. 1.9.1 (Peterson 2021), DHARMa v. 0.4.7 (Hartig 2022), ecospat v. 4.1.3 (Broennimann et al. 2024), emmeans v. 2.0.2 (Lenth 2023), ggplot2 v. 4.0.2 (Wickham 2016), glmmTMB v. 1.1.5 (Brooks et al. 2017), indicspecies v. 1.7.12 (De Cáceres and Legendre 2009) and vegan v. 2.6.10 (Oksanen et al. 2025).

## Results

### Climatic filtering shapes the ultramafic symbiosis

**Figure 3.**
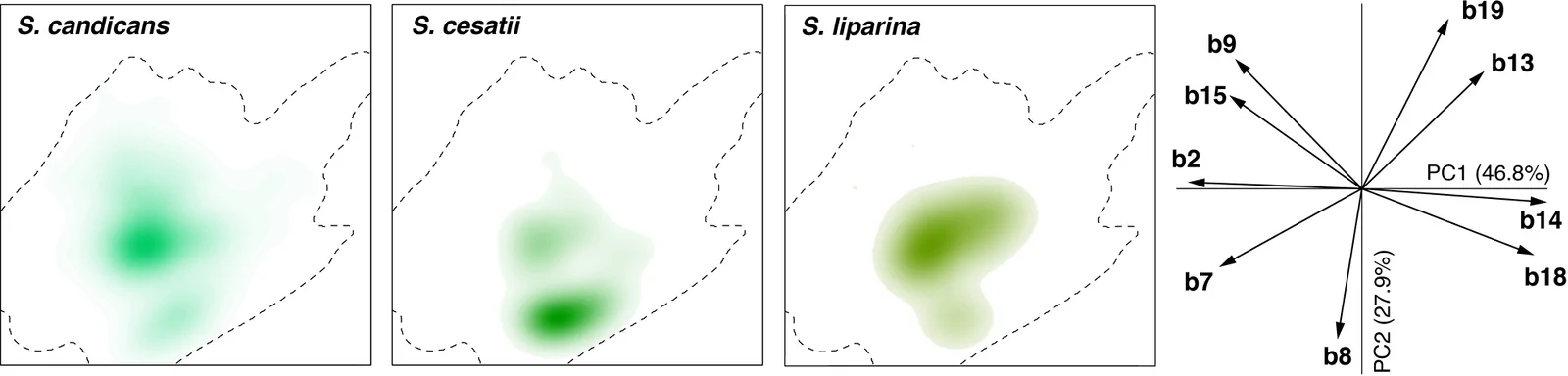
Climatic space of *S. liparina* in the comparison with the studied congeners. Differences in environmental niches of the three *Solenopsora* lichen species are shown in principal component space as kernel density maps, with shading proportional to occurrence density. Dashed outlines delimit the available environmental space. The right panel is a PCA plot showing environmental variable loadings on the first two components. Variance explained by each component is given in parentheses. b2 = Mean Diurnal Range, b7 = Temperature Annual Range, b8 = Mean Temperature of Wettest Quarter, b9 = Mean Temperature of Driest Quarter, b13 = Precipitation of Wettest Month, b14 = Precipitation of Driest Month, b15 = Precipitation Seasonality, b18 = Precipitation of Warmest Quarter, b19 = Precipitation of Coldest Quarter.

Phylogenetic inference using RAxML recovered *S. liparina* as a well-defined monophyletic taxon. *Solenopsora liparina* formed a clade sister to the *S. candicans* and *Solenopsora* sp. 1 lineage, while *S. cesatii* was recovered as sister to the clade comprising *S. liparina*, *S. candicans*, and *Solenopsora* sp. 1 (Suppl. material 1: fig. S2). Climatic niche modelling of *S. liparina* identified a core area of high suitability (>0.75) concentrated in the northern Mediterranean (Fig. 2), whereas the high suitability for the two congeners *S. candicans* and *S. cesatii* is in the Mediterranean-Atlantic and oro-Mediterranean, respectively (Suppl. material 1: figs S3, S4). Precipitation during both the wettest and driest quarters, as well as during the coldest part of the year (Suppl. material 1: figs S5–S7, Suppl. material 3: table S5), structured the potential climatic niche of *S. liparina*, indicating that the species occupies a restricted climatic envelope defined by moisture availability during periods of physiological activity. Consistent with this pattern, PCA environmental space showed that *S. liparina* occupies a narrower and partially distinct position relative to its calcicolous congeners (Suppl. material 1: figs S8–S10, Suppl. material 3: table S6), primarily along gradients associated with precipitation variables (BIO13, BIO19; Fig. 3), suggesting a shift toward wetter winter conditions within a broadly overlapping climatic space. This climatic positioning is consistent with MaxEnt variable importance and response curves (Suppl. material 1: figs S5–S10, Suppl. material 3: table S5), which identify the precipitation variables, particularly BIO13 and BIO19, as key determinants of habitat suitability in *S. liparina* (Suppl. material 1: figs S5–S7, Suppl. material 3: table S5). Niche equivalency and similarity tests indicated no significant niche divergence between *S. liparina* and *S. candicans* or *S. cesatii* (Suppl. material 3: table S6), with the exception of the comparison between *S. candicans* and *S. cesatii* in the equivalency test. The results suggest that not all ultramafic habitats are climatically equivalent for *S. liparina*, with precipitation-related variables further restricting the range of substrate conditions within its substrate-defined niche.

### *Symbiochloris*-dominated photobiont assemblages exhibit host-associated and climate-structured turnover

**Figure 4.**
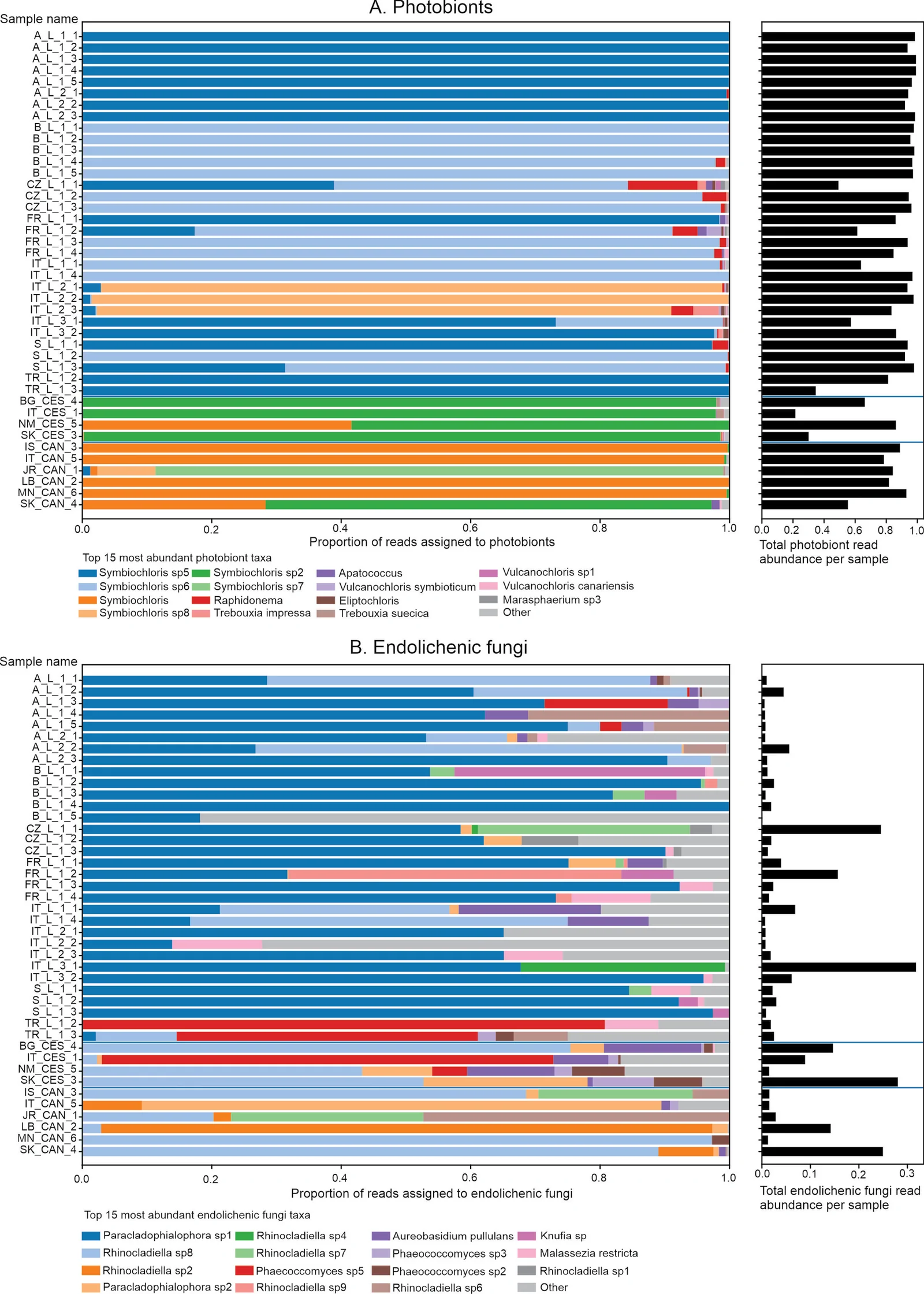
Relative proportion of reads and total read abundance of **A** photobionts and **B** endolichenic fungi. Y-axis labels indicate *Solenopsora* species abbreviations: L – *S. liparina*, CES – *S. cesatii*, CAN – *S. candicans*.

Metabarcoding data showed that *S. liparina* thalli were dominated by the mycobiont, with non-mycobiont reads primarily representing *Chlorophyta* photobionts (all within class *Trebouxiophyceae*) and additional *Ascomycota*, whereas other taxonomic groups contributed only marginally to total read abundance (Suppl. material 1: fig. S11). Photobiont assemblages were OTU-rich but strongly uneven (Fig. 4A). For details on taxonomic assignments of OTUs, functional guild composition, and relative read abundance, see Suppl. material 1: fig. S12 and Suppl. material 4: tables S7–S13. Within thalli, photobiont assemblages were most often dominated by a single lineage of the genus *Symbiochloris* contributing nearly all reads, whereas additional lineages, when present, occurred at low relative abundance, indicating low-frequency co-occurrence rather than balanced multi-lineage symbioses (Fig. 4A, Suppl. material 1: figs S13, S14, Suppl. material 4: tables S13, S17). In the context of calcicolous congeners, the composition of dominant photobionts of *S. liparina* showed a shift, with two *Symbiochloris* lineages restricted to *S. liparina* and two linked to calcicolous hosts, while low-abundance lineages were broadly shared among host lichens (Suppl. material 1: fig. S13). This pattern is consistent with the prediction that substrate-related effects can be expressed primarily through shifts in the relative frequency of symbiont lineages rather than complete lineage segregation.

**Figure 5.**
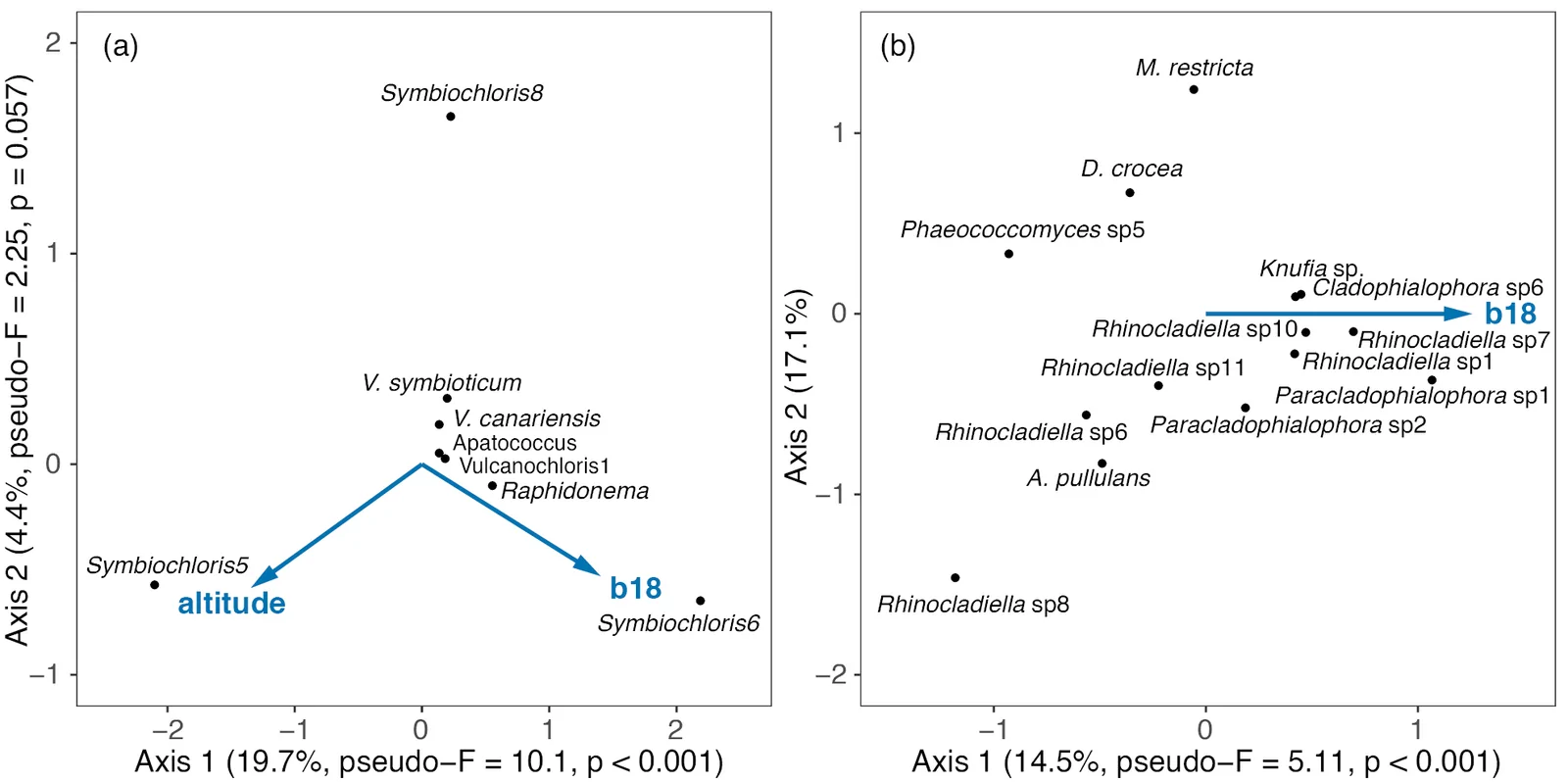
Significant relationships between environmental variables and community composition of photobionts (**a**) and endolichenic fungi (**b**) of *S. liparina*. Arrows depict fitted environmental vectors and points are species scores for the top 25% best-fitting taxa. Percent variation explained by each axis, pseudo-F statistics, and associated p-values are shown in parentheses. b18 = Precipitation of Warmest Quarter.

Variation in photobiont community composition was significantly structured by precipitation during the warmest quarter (BIO18), with altitude additionally contributing to observed patterns (Suppl. material 4: table S16, Fig. 5A), indicating climate-driven lineage turnover across environmental gradients. Elevated diversity and evenness were rare and occurred only in a subset of the samples from Central European and Atlantic populations, followed by samples from the Apennine Peninsula and Serbia (Suppl. material 1: fig. S13, Suppl. material 4: table S17). At the population level, no statistically significant differences were detected for any diversity index (Suppl. material 1: fig. S13). Most of the alpha diversity metrics were largely insensitive to environmental predictors, with only photobiont richness responding to annual temperature range (Suppl. material 1: fig. S15, Suppl. material 4: table S16). These results support the prediction that photobiont community composition is primarily structured by climatic gradients, whereas alpha diversity exhibits comparatively weak environmental response.

Phylogenetic reconstructions of photobionts inferred from *rbcL* and ITS (ITS1–5.8S–ITS2) datasets under maximum likelihood frameworks corroborated metabarcoding-derived patterns, revealing diversity within the genus *Symbiochloris*, including two previously unrecognized clades (Suppl. material 2: figs S16–S18). Most Sanger-derived ITS2 sequences corresponded to the most abundant OTUs recovered from metabarcoding data, confirming their dominance across samples.

### Accessory fungal partners show environmentally structured community composition

Endolichenic fungi contributed only a minor fraction of the holobiont diversity. For details on taxonomic assignments, functional guild composition, and relative read abundance, see Suppl. material 1: fig. S12 and Suppl. material 3, 4: tables S7–S12, S14. Endolichenic fungi exhibited high diversity and evenness, considerable intra-population variability, and low dominance values (Fig. 4B, Suppl. material 2: fig. S19, Suppl. material 4: table S17). Notably, one taxon (*Paracladophialophora* sp. 1) was consistently shared across all *S. liparina* samples, while another (*Rhinocladiella* sp. 8) was shared across all but one sample of the calcicolous congeners. Other endolichenic fungal lineages were broadly distributed across *S. liparina* and its calcicolous congeners, with many lineages shared among host taxa and mostly exhibiting no clear host-specific pattern (Suppl. material 2: fig. S20). The environmental predictor BIO18 (precipitation of warmest quarter) explained variation in their community composition but was not associated with any alpha diversity metrics, indicating environmentally driven lineage turnover rather than changes in overall diversity (Suppl. material 2: fig. S19, Suppl. material 4: table S16, Fig. 5B). These findings suggest that endolichenic fungal community composition varies primarily along climatic gradients, whereas alpha diversity remains comparatively insensitive to environmental variation.

## Discussion

Our study reveals distinct patterns of diversity and environmental responsiveness for the studied symbiotic partners of the ultramafic lichen specialist *Solenopsora liparina*, a well-defined, monophyletic species (cf. Guttová et al. 2014; Fačkovcová et al. 2020; Suppl. material 1: fig. S2). Lichen thalli were typically dominated by a single *Symbiochloris* lineage (Fig. 4A, Suppl. material 1: fig. S14, Suppl. material 4: tables S13, S17), consistent with patterns reported in other representatives of *Catillariaceae* (Gheza et al. 2025), *Coniocybaceae*, *Lobariaceae* or *Ramalinaceae* (Sanders and Masumoto 2021). In addition, low-abundance algal lineages from multiple genera were detected (Suppl. material 1: fig. S14), reflecting photobiont plurality within individual thalli (cf. Dědková et al. 2025). Photobiont and endolichenic fungal assemblages were primarily structured by climatic variables, particularly precipitation and, to a lesser extent, altitude (Suppl. material 4: table S16, Fig. 5A). This indicates that climatic filtering contributes to structuring the photobiont community (cf. Dal Grande et al. 2018; Medeiros et al. 2021). Because substrate identity was not explicitly included as an independent predictor in the statistical models, any substrate-associated interpretation is based on comparisons among host taxa rather than direct testing. Substrate chemistry appears to influence photobiont associations primarily by shaping the composition of dominant partners, while minor taxa are broadly shared across hosts. Together, these patterns are consistent with a model of mixed host-dependence, in which dominant photobionts may exhibit host- or substrate-linked preferences, whereas minor (less abundant) associates show weaker specificity and broader ecological overlap. Endolichenic fungi appeared to exhibit higher species richness, greater evenness, and higher intra-population variability than photobionts (Fig. 4B, Suppl. material 2: figs S19, S20, Suppl. material 4: table S17). Their community composition responds primarily to precipitation patterns, reflecting environmentally driven lineage turnover. Widespread occurrence, combined with the prevalent absence of strong host partitioning, suggests that endolichenic fungal communities can be weakly structured by substrate chemistry but responsive to climatic gradients.

### Climatic filtering and selective gradients across interacting lineages

The climatic profile of *S. liparina* demonstrates that substrate specialisation alone does not determine its realized distribution. Precipitation during physiologically critical periods, particularly during the warmest and coldest quarters, emerges as a dominant predictor of lichen occurrence (Ellis 2019). This pattern suggests that the joint influence of chronic edaphic stress and recurrent climatic stress may contribute to maintaining traits linked to substrate tolerance, while still allowing ecological sorting among more susceptible symbiotic partners. Among congeners, *S. liparina* exhibits the most consistent precipitation-driven signal in its MaxEnt variable-importance profile, whereas *S. candicans* and *S. cesatii* profiles rely more on temperature-related predictors and precipitation seasonality. This contrast suggests that ultramafic specialisation may be associated with a narrower climatic niche relative to calcicolous congeners, as reflected by the stronger dependence of *S. liparina* on precipitation-related variables. Such a pattern is consistent with the ecological filtering imposed by ultramafic substrates, although direct tests of niche evolution and diversification dynamics would be required to assess whether this pattern reflects long-term evolutionary constraints or simply stronger ecological filtering associated with ultramafic habitats. The apparent discrepancy between the low habitat suitability predicted across much of the Central European range of *S. candicans* and its observed occurrence is likely attributable to the position of these populations at the climatic margins of the species‘ distribution, where continental climatic conditions coincide with a limited availability of suitable habitats. A previous study (Fačkovcová et al. 2017) demonstrated strong niche conservatism in *S. candicans* across its distribution range, suggesting that populations occurring in Central Europe occupy ecological conditions similar to those in Mediterranean core areas rather than exhibiting niche shifts at the range margin. Consequently, the restricted and fragmented distribution of suitable habitats in Central Europe may explain the persistence of isolated populations despite generally low climatic suitability predicted by broad-scale models.

The distinct phylogenetic position and long independent evolutionary history of *S. liparina* inferred previously from time-calibrated analyses (Fačkovcová et al. 2020) indicate that ultramafic specialisation has been maintained over extended evolutionary timescales. Nevertheless, in this study we do not explicitly test rates of niche evolution or diversification, and therefore any inference regarding evolutionary constraint should be regarded as tentative. This interpretation is also supported by the ecological association of *S. liparina* for habitats characterized by elevated temperatures during the driest quarter, suggesting adaptation to the warmer and drier conditions associated with ultramafic bedrock. Indeed, the darker colour of ultramafic rocks results in higher heat conductivity and greater heat absorption compared to the lighter limestones or dolomites, thus making these habitats more challenging for survival (Konečná et al. 2020). Based on our field observations, *S. liparina* frequently occurs at sites receiving partial shading from nearby trees and shrubs, which may locally buffer substrate temperatures and reduce desiccation stress on ultramafic outcrops. At the same time, the ecological optimum of *S. liparina* is also linked to increased winter precipitation, which may help buffer against extreme summer climatic conditions.

Nonetheless, we presume that the distribution and survival of this ultramafic specialist are also strongly influenced by microclimatic refugia, such as sheltered rock fissures and crevices or deep, moist valleys (Kučera et al. 2010; Fačkovcová et al. 2020). These microhabitats may buffer harsh environmental conditions and facilitate the persistence of *S. liparina* even in drier, warmer parts of its distribution range. However, this hypothesis requires further comparative investigation of microclimatic conditions at the microscale. Microhabitat refugia, including rock fissures and shaded surfaces, likely play a key bilising role, sustaining symbiotic function in otherwise hostile environments (Jung and Büdel 2021). The climate sensitivity of the associated microbiome further indicates that ultramafic lichen holobionts are structured by the combined effects of substrate and climate rather than substrate alone.

### Climate- and substrate-responsive lineage turnover in photobionts

Photobiont communities associated with *Solenopsora liparina* display clear environmental structuring that varies along gradients of warm-season precipitation and altitude (Fig. 5A). These patterns reflect climate-associated turnover. This, together with extreme thermal properties of ultramafic substrates, likely limit the distribution and survival of entire lichen (cf. Favero-Longo et al. 2004; Medeiros et al. 2021). Photobiont species richness declines with increasing annual temperature range (Suppl. material 1: fig. S15), whereas diversity, evenness, and dominance patterns remain largely insensitive to environmental predictors, indicating stability in abundance structure despite compositional turnover. Together with evidence for shared and putatively host-associated photobiont lineages, these patterns suggest climate-associated turnover in photobiont communities driven by environmental filtering from a broadly distributed pool. Host dependence patterns may appear to vary among lineages rather than reflecting strict host-driven exclusivity.

The photobiont composition of *S. liparina* compared to calcicolous congeners indicates a shift in major photobionts but sharing of the minor ones (Suppl. material 1: fig. S14). This suggests that substrate chemistry may selectively influence dominant photobionts, while low-abundance lineages remain broadly shared among hosts. However, the observed trend in major photobionts needs to be interpreted with caution due to the small sample size of calcicolous congeners, although samples encompass the ecological breadth of their distribution ranges. Comparable low substrate specificity has been observed in globally distributed lichen species (Muggia et al. 2014), emphasizing the general ecological flexibility of photobionts rather than the absence of specialisation. Similarly, certain soil algae and cyanobacteria on mafic and ultramafic substrates have been shown to tolerate high levels of heavy metals, forming similar clades on both metal-rich and unpolluted substrates (Bačkor et al. 2010).

In addition to major *Symbiochloris* photobionts, *S. liparina* thalli include a range of *Trebouxia* taxa – the most common algal genus in lichens (cf. Dědková et al. 2025), reflecting access to a shared pool of generalist photobionts. Photobiont plurality, i.e. the occurrence of multiple photobiont lineages within a single lichen symbiosis, has been shown to decline in climatically stressful environments, often interpreted as functional redundancy and symbiotic flexibility (Tagirdzhanova et al. 2025), without compromising host specificity (Mark et al. 2020). In contrast, chemically stressful or toxic substrates may maintain or even promote photobiont diversity (Bačkor et al. 2010; Osyczka et al. 2020). Alternatively, photobiont plurality may represent a pool of functionally distinct partners, from which the mycobiont selectively associates with the physiologically most efficient lineages, enabling rapid physiological adjustment without genetic change (Casano et al. 2011).

Our data support earlier findings that warm, dry, and climatically variable conditions tend to reduce photobiont plurality and reinforce dominance by well-adapted lineages (De Carolis et al. 2022). Richness negatively correlates with annual temperature range, highlighting the importance of thermal stability. We found no consistent effect of altitude on richness, suggesting that climatic variability rather than elevation per se drives these patterns, while overall photobiont community composition is structured by precipitation and altitude. While lichen hyphae may buffer photobionts from substrate toxicity through metal binding and chelation (Bačkor and Fahselt 2008), our results indicate that climate is a key driver of photobiont species composition and turnover, whereas substrate-associated differences appear limited to dominant partners when compared with calcicolous congeners. This emphasizes that environmental filtering primarily shapes community composition under suboptimal climatic conditions, while substrate conditions influence which photobiont taxa dominate within those communities. These findings align with previous studies showing that lichen photobionts generally exhibit broad ecological amplitude and functional flexibility across extreme environments, where strong environmental filters govern symbiotic specificity (Bačkor et al. 2010; Blázquez et al. 2022; Fernández-Mendoza et al. 2011; Pérez-Ortega et al. 2012; Determeyer-Wiedmann et al. 2019; Osyczka et al. 2020; De Carolis et al. 2022; Li et al. 2024). Similarly, climatic stress has been shown to reduce intra-thalline plurality and favour dominance by a single lineage (Peksa and Škaloud 2011; Škvorová et al. 2022), consistent with our observations in Eastern Mediterranean ultramafic sites. *Solenopsora liparina* associates with multiple phylogenetically distinct photobiont lineages from a broadly distributed pool, with local community composition shaped primarily by climatic conditions.

### Moisture-driven assembly of endolichenic fungi

Endolichenic fungi show compositional shifts along the warm-season precipitation gradient, consistent with the effect of precipitation of the warmest quarter on community composition. Unlike soil fungal communities structured by geographic separation (Gourmelon et al. 2016), endolichenic fungal assemblages in *S. liparina* vary more within than among populations. This pattern reflects high intra-population variability and weak population-level differentiation, suggesting the fine-scale environmental filtering may override dispersal limitation, leading to fine-scale community genetic structuring within thalli, i.e. non-random distribution of genetic variation among co-occurring symbiont lineages.

Many melanized lineages, including dark septate endophytes in the genera *Cladophialophora* and *Rhinocladiella*, known for extremotolerance (Harutyunyan et al. 2008; Selbmann et al. 2013; Muggia et al. 2016; Smith et al. 2020), occur across samples of *S. liparina* spanning the precipitation gradient, with their relative abundance varying among samples rather than showing a uniform directional response. This pattern is in line with lineage-specific response to environmental conditions, although direct physiological interpretation remains tentative. Community turnover along this gradient likely reflects differential ecological responses among lineages rather than uniform environmental filtering. In contrast, *Paracladophialophora* sp. 1 exhibited an interesting distribution pattern, being highly frequent in the ultramafic specialist *S. liparina* while remaining undetected in the calcicolous congeners. The observation is noteworthy because the genus *Paracladophialophora* belongs to the order *Chaetothyriales*, which includes numerous melanized fungi frequently associated with stress-tolerant lifestyles and extreme habitats (Cometto et al. 2023). However, the limited sampling of calcicolous species precludes a formal assessment of substrate specificity or host preference. Consequently, whether the predominance of *Paracladophialophora* sp. 1 reflects adaptation to ultramafic-associated conditions, host-related factors, or simply incomplete sampling remains unresolved and warrants further investigation.

The absence of strong geographic structure combined with climatic sensitivity suggests that endolichenic fungi function as dynamically assembled communities responding to environmental variation. Summer hydration events have been shown to directly influence the carbon balance and physiological performance of primary symbionts, and to create pronounced asymmetries in symbiont activity (Meyer et al. 2024), suggesting that endolichenic fungi may also be indirectly influenced by moisture-driven dynamics. Accurate inference of guild composition and functional roles further requires careful sequence curation and cross-validation (Muggia and Grube 2018).

## Conclusions

Ultramafic ecosystems provide natural laboratories for studying adaptation under extreme edaphic stress. In the ultramafic specialist lichen *S. liparina*, symbiotic organisation is shaped by climatic filtering within a specialised edaphic niche, with climate acting as a consistent structuring correlate across symbiont groups. Photobionts and endolichenic fungi exhibit climate-associated compositional turnover. Alpha diversity patterns appear to differ between studied symbiont groups and are generally less responsive than community composition. In our dataset, photobionts show indications of substrate- or host-associated structuring, reflected in differences in the most frequently detected lineages between *S. liparina* and the comparatively limited set of sampled calcicolous congeners, whereas less frequent lineages are shared across host systems. Endolichenic fungi show weaker and less consistent host-associated patterns, suggesting that climate factors may play a larger role in structuring communities across symbiont groups. These findings indicate that persistence in ultramafic environments relies on flexible, compositionally dynamic symbioses rather than highly specific obligate partnerships. Climatic filtering plays an important role in shaping symbiotic composition, suggesting that environmentally responsive partner assembly may contribute to persistence in extreme habitats.

## Additional information

### Artificial Intelligence (AI) use

The authors accept full responsibility for the content of the manuscript, including the disclosure of any use of AI.

No AI tools were used in the preparation of this manuscript.

### Adherence to national and international regulations

All the fungal strains used in this study have been legally obtained, respecting the Convention on Biological Diversity (Rio Convention).

### Author ORCIDs

M. Caboň https://orcid.org/0000-0002-2255-3816

M. Slovák https://orcid.org/0000-0001-9917-3099

M. Svitok https://orcid.org/0000-0003-2710-8102

J. Steinová https://orcid.org/0000-0003-0229-4535

Z. Fačkovcová https://orcid.org/0000-0002-5760-5364

J. Kučera https://orcid.org/0000-0002-9983-7630

L. Paoli https://orcid.org/0000-0001-5479-6428

T. Větrovský https://orcid.org/0000-0002-0831-486X

A. Hindáková https://orcid.org/0000-0003-1445-5236

D. Senko https://orcid.org/0000-0002-0630-043X

J. Zozomová-Lihová https://orcid.org/0000-0002-8950-6643

A. Melichárková https://orcid.org/0000-0003-4986-3774

Z. Gajdošová https://orcid.org/0000-0002-1395-1070

A. Guttová https://orcid.org/0000-0001-9394-1077

### Data availability

Newly generated sequences are deposited in the GenBank database under accession numbers listed in Suppl. material 4: table S15. Metabarcoding data are deposited in NCBI SRA PRJNA1392552. All sequence alignments are deposited in Figshare at [doi: 10.6084/m9.figshare.31081510]. All associated information is available in the Suppl. materials.

## Supplementary materials

Supplementary material 1 Additional figures S1–S15 Data type docx https://binary.pensoft.net/file/1753397This dataset is made available under the Open Database License (http://opendatacommons.org/licenses/odbl/1.0/). The Open Database License (ODbL) is a license agreement intended to allow users to freely share, modify, and use this Dataset while maintaining this same freedom for others, provided that the original source and author(s) are credited. Miroslav Caboň, Marek Slovák, Marek Svitok, Jana Steinová, Zuzana Fačkovcová, Jaroslava Gužiková, Jaromír Kučera, Luca Paoli, Tomáš Větrovský, Alica Hindáková, Dušan Senko, Judita Zozomová-Lihová, Andrea Melichárková, Zuzana Gajdošová, Anna Guttová

Supplementary material 2 Additional figures S16–S20 Data type zip https://binary.pensoft.net/file/1753398This dataset is made available under the Open Database License (http://opendatacommons.org/licenses/odbl/1.0/). The Open Database License (ODbL) is a license agreement intended to allow users to freely share, modify, and use this Dataset while maintaining this same freedom for others, provided that the original source and author(s) are credited. Miroslav Caboň, Marek Slovák, Marek Svitok, Jana Steinová, Zuzana Fačkovcová, Jaroslava Gužiková, Jaromír Kučera, Luca Paoli, Tomáš Větrovský, Alica Hindáková, Dušan Senko, Judita Zozomová-Lihová, Andrea Melichárková, Zuzana Gajdošová, Anna Guttová

Supplementary material 3 Additional tables S1–S9 Data type docx https://binary.pensoft.net/file/1753399This dataset is made available under the Open Database License (http://opendatacommons.org/licenses/odbl/1.0/). The Open Database License (ODbL) is a license agreement intended to allow users to freely share, modify, and use this Dataset while maintaining this same freedom for others, provided that the original source and author(s) are credited. Miroslav Caboň, Marek Slovák, Marek Svitok, Jana Steinová, Zuzana Fačkovcová, Jaroslava Gužiková, Jaromír Kučera, Luca Paoli, Tomáš Větrovský, Alica Hindáková, Dušan Senko, Judita Zozomová-Lihová, Andrea Melichárková, Zuzana Gajdošová, Anna Guttová

Supplementary material 4 Additional tables S10–S17 Data type xlsx https://binary.pensoft.net/file/1753400This dataset is made available under the Open Database License (http://opendatacommons.org/licenses/odbl/1.0/). The Open Database License (ODbL) is a license agreement intended to allow users to freely share, modify, and use this Dataset while maintaining this same freedom for others, provided that the original source and author(s) are credited. Miroslav Caboň, Marek Slovák, Marek Svitok, Jana Steinová, Zuzana Fačkovcová, Jaroslava Gužiková, Jaromír Kučera, Luca Paoli, Tomáš Větrovský, Alica Hindáková, Dušan Senko, Judita Zozomová-Lihová, Andrea Melichárková, Zuzana Gajdošová, Anna Guttová

Supplementary material 5 Captions for the additional figures and tables Data type docx https://binary.pensoft.net/file/1753401This dataset is made available under the Open Database License (http://opendatacommons.org/licenses/odbl/1.0/). The Open Database License (ODbL) is a license agreement intended to allow users to freely share, modify, and use this Dataset while maintaining this same freedom for others, provided that the original source and author(s) are credited. Miroslav Caboň, Marek Slovák, Marek Svitok, Jana Steinová, Zuzana Fačkovcová, Jaroslava Gužiková, Jaromír Kučera, Luca Paoli, Tomáš Větrovský, Alica Hindáková, Dušan Senko, Judita Zozomová-Lihová, Andrea Melichárková, Zuzana Gajdošová, Anna Guttová
