# Supplementary material for: Ultramafic specialist lichens persist through flexible symbioses structured by climate-driven filtering and modulated by substrate effects
Source: IMA Fungus. 2026 Aug 24;17:e199447. doi: 10.3897/imafungus.17.199447 (PMC13531026; doi:10.3897/imafungus.17.199447)
Supplement: Supplementary file 1 [file imafungus-17-e199447-s001.docx]

**Ultramafic specialist lichens persist through flexible symbioses structured by climate-driven filtering and modulated by substrate effects**

**Miroslav Caboň^1^, Marek Slovák^1,^**^2,^***, Marek Svitok^1,3,4^, Jana Steinová^5^, Zuzana Fačkovcová^1^**, **Jaroslava Gužiková^6^, Jaromír Kučera^1^, Luca Paoli^7^,** **Tomáš Větrovský^8^, Alica Hindáková^1^, Dušan Senko^1^, Judita Zozomová-Lihová^1^, Andrea Melichárková^1^, Zuzana Gajdošová^1^, Anna Guttová^1^**

^1^Plant Science and Biodiversity Centre, Slovak Academy of Sciences, Dúbravská cesta 9, 845 23 Bratislava, Slovakia

^2^Department of Botany, Faculty of Science, Charles University, 128 01 Prague, Czech Republic

^3^Department of Biology and General Ecology, Faculty of Ecology and Environmental Sciences, Technical University in Zvolen, 960 01 Zvolen, Slovakia

^4^Department of Forest Ecology, Faculty of Forestry and Wood Sciences, Czech University of Life Sciences Prague, Kamýcka 129, Prague CZ – 165 21, Czech Republic

^5^Department of Botany, Faculty of Science, Charles University in Prague, Benátská 2, 128 01 Prague, Czech Republic

^6^Comenius University Bratislava, Jessenius Faculty of Medicine in Martin, Malá Hora 4, 03601 Martin, Slovakia

^7^Department of Biology, University of Pisa, via Ghini 13, 56126 Pisa, Italy

^8^Institute of Microbiology, Academy of Sciences of the Czech Republic, Vídeňská 1083, 142 00 Prague, Czech Republic

*corresponding author. marek.slovak@savba.sk

**Supplementary material – figures S1–S15**

**Figure S1.** Analytical workflow.

**Fig. S2.** Maximum likelihood phylogeny of studied *Solenopsora* species inferred from concatenated sequences (ITS, KS and *mcm7*) using RAxML under GTR+G model. Node support values are shown as ML bootstrap percentages based on 1000 replicates / Bayesian posterior probabilities. The scale bar represents substitutions per sites. For sample codes of sequences generated for this study see Table S3, other sample codes follow Guttová et al. 2014.

**Fig. S3.** Projection of recent HSM of *Solenopsora candicans* across its range. Colours indicate the predicted habitat suitability ranging from 0.25 (low suitability) to 1.0 (high suitability). Black dots represent known occurrence records of *S. candicans*. Light green shading indicates predicted habitat suitability values < 0.25, which fall below the range represented by the colour scale in the figure legend.

**Fig. S4.** Projection of recent HSM of *Solenopsora cesatii* across its range. Colours indicate the predicted habitat suitability ranging from 0.25 (low suitability) to 1.0 (high suitability). Black dots represent known occurrence records of *S. cesatii*.

**Fig. S5.** Predictive estimate of importance of variables used for MaxEnt models – results of Jacknife test for training data of *Solenopsora liparina*. b2 = Mean Diurnal Range, b7 = Temperature Annual Range, b8 = Mean Temperature of Wettest Quarter, b9 = Mean Temperature of Driest Quarter, b13 = Precipitation of Wettest Month, b14 = Precipitation of Driest Month, b15 = Precipitation Seasonality, b18 = Precipitation of Warmest Quarter, b19 = Precipitation of Coldest Quarter.

**Fig. S6.** Predictive estimate of importance of variables used for MaxEnt models – results of Jacknife test for training data of *Solenopsora candicans*. b2 = Mean Diurnal Range, b7 = Temperature Annual Range, b8 = Mean Temperature of Wettest Quarter, b9 = Mean Temperature of Driest Quarter, b13 = Precipitation of Wettest Month, b14 = Precipitation of Driest Month, b15 = Precipitation Seasonality, b18 = Precipitation of Warmest Quarter, b19 = Precipitation of Coldest Quarter.

**Fig. S7.** Predictive estimate of importance of variables used for MaxEnt models – results of Jacknife test for training data of *Solenopsora cesatii*. b2 = Mean Diurnal Range, b7 = Temperature Annual Range, b8 = Mean Temperature of Wettest Quarter, b9 = Mean Temperature of Driest Quarter, b13 = Precipitation of Wettest Month, b14 = Precipitation of Driest Month, b15 = Precipitation Seasonality, b18 = Precipitation of Warmest Quarter, b19 = Precipitation of Coldest Quarter.

**Fig. S8.** Response curves demonstrating how only the corresponding variable contributes to the HSM of *S. liparina*. b2 = Mean Diurnal Range, b7 = Temperature Annual Range, b8 = Mean Temperature of Wettest Quarter, b9 = Mean Temperature of Driest Quarter, b13 = Precipitation of Wettest Month, b14 = Precipitation of Driest Month, b15 = Precipitation Seasonality, b18 = Precipitation of Warmest Quarter, b19 = Precipitation of Coldest Quarter.

| a)   | b) |
| --- | --- |
| c)   | d)   |
| e)   | f)   |
| g)   | h)   |
| i)   |  |

**Fig. S9.** Response curves demonstrating how only the corresponding variable contributes to the HSM of *S. candicans*. b2 = Mean Diurnal Range, b7 = Temperature Annual Range, b8 = Mean Temperature of Wettest Quarter, b9 = Mean Temperature of Driest Quarter, b13 = Precipitation of Wettest Month, b14 = Precipitation of Driest Month, b15 = Precipitation Seasonality, b18 = Precipitation of Warmest Quarter, b19 = Precipitation of Coldest Quarter.

| a)   | b)   |
| --- | --- |
| c)   | d)   |
| e)   | f)   |
| g)   | h)   |
| i)   |  |

**Fig. S10.** Response curves demonstrating how only the corresponding variable contributes to the HSM of *S. cesatii*. b2 = Mean Diurnal Range, b7 = Temperature Annual Range, b8 = Mean Temperature of Wettest Quarter, b9 = Mean Temperature of Driest Quarter, b13 = Precipitation of Wettest Month, b14 = Precipitation of Driest Month, b15 = Precipitation Seasonality, b18 = Precipitation of Warmest Quarter, b19 = Precipitation of Coldest Quarter.

| a)   | b)   |
| --- | --- |
| c)   | d)   |
| e)   | f)   |
| g)   | h) |
| i)   |  |

**Fig. S11.** Taxonomic identification of OTUs to the phylum level per samples after removing the primary mycobiont reads. Values represent relative read abundance based on amplicon sequencing data for each sample. For sample codes see Table S3.

**Fig. S12.** Functional guilds per samples after removing the primary mycobiont reads. Values are based on amplicon sequencing data. For sample codes see Table S3. EcM – ectomycorrhizal fungi, ND – not determined.

**Fig. S13.** Diversity metrics of photobiont communities across populations of S. liparina and number of recognized photobiont OTUs. Boxplots show the distribution of six diversity indices: Shannon Index, Inverse Simpson Index, Simpson 1D Index, Dominance Index, and Evenness Index, for each sampled population. The central line indicates the median, boxes the interquartile range (IQR), and whiskers value within 1.5 × IQR. Letters above the boxes indicate groupings based on Tukey’s HSD post-hoc test, with shared letters denoting no significant difference among populations within each metric. Details on sample codes are given in Table S3.

**Fig. S14.** Relative abundance reads of endolichenic photobionts. For sample codes see Table S3. Bubble colour corresponds to the studied *Solenopsora* species as follows: LIP – *S. liparina*, CES – *S. cesatii*, CAN – *S. candicans*.

**Figure S15.** Significant relationship between the temperature annual range (b7) and species richness of *S*. *liparina* photobionts. Predictions from a Poisson GLMM (solid line) are shown with 95% confidence intervals (shaded band).
