# Supplementary material for: Ultramafic specialist lichens persist through flexible symbioses structured by climate-driven filtering and modulated by substrate effects
Source: IMA Fungus. 2026 Aug 24;17:e199447. doi: 10.3897/imafungus.17.199447 (PMC13531026; doi:10.3897/imafungus.17.199447)
Supplement: Supplementary file 2 [file imafungus-17-e199447-s003.docx]

**Supporting information – Tables S1–S9**

**Table S1.** List of occurrence data for *Solenopsora liparina* based on revised specimens and own field study. Information on locality comprises data as follows: country, the nearest town/village/name of the place, altitude in m a. s. l., collectors' abbreviations (complete list given in the caption of Table S 1), and year. Information on collection includes abbreviation of herbarium according to Index Herbariorum (https://sweetgum.nybg.org/science/ih/) with the code/number of specimens, if available. Geographical position (latitude and longitude) is given.

Collectors abbreviations: AG – A. Guttová (Bérešová); CS – C. Sbarbaro; EŠ - E. Štubňová; ET - E. Timdal; FBG – F. Baglietto; JK - J. Kučera; KO - ; JPH - J. P. Halda; JV - J. Vondrák; ; LL – L. Lőkös; LP - L. Paoli; KP - K. Piknová; MCA - M. Candan; MS - M. Slovák; MV - M. Valachovič; MZ - M. Zmrhalová; KO – K. Olšavská; PWJ – P. W. James; RT - R. Tsonev; TP - T. Pisani; ZF - Z. Fačkovcová.

|  | **Taxon** | **Locality** | **Collection** | **lat** | **long** |
| --- | --- | --- | --- | --- | --- |
| 1 | S. liparina | Albania, Mollas, 910 m, AG & MS, 2021 | SAV0010610 | 40.4619 | 20.6675 |
| 2 | S. liparina | Albania, Hudënisht, 700 m, AG & MS, 2021 | SAV0010608 | 40.9828 | 20.6394 |
| 3 | S. liparina | Albania, Piskupat, 700 m, AG & MS, 2021 | SAV0010601 | 41.0205 | 20.6361 |
| 4 | S. liparina | Albania,Sofra e Skenderbeut (Guri i Pishkashit), Pishkash, ca 930 m, LL, 2016 | BP 96256 | 41.0982 | 20.5158 |
| 5 | S. liparina | Albania, Elbasan County (Qarku i Elbasanit), Ura e Murrashit, Mirakë - Librazhd, ca 330 m, LL, 2016 | BP 96255 | 41.1838 | 20.2759 |
| 6 | S. liparina | Bosna and Hercegovina, Smolin Mts, Milankovići, Krivaja 450 m, AG & MS, 2021 | SAV0010603 | 44.2230 | 18.4884 |
| 7 | S. liparina | Czech Republic, Raškov, Vysoký kámen, ca 550–600 m, AV, 1970; ibid., 528 m, AG, LP, JPH & MZ, 2011 | PRA-V-01981, SAV0010532 | 50.0447 | 16.8877 |
| 8 | S. liparina | France, Limousine, Haute Vienne, Magnac Bourg, La lande de la Flotte et du Cluzeau, 440 m, AG & LP, 2012 | SAV0010534 | 45.5828 | 1.3955 |
| 9 | S. liparina | France, Limousine, Haute Vienne, La Roche – l´Abeille, La lande de Saint-Laurent, 400 m, AG & LP, 2012 | H-NYL29346, SAV0010540 | 45.6070 | 1.2474 |
| 10 | S. liparina | Great Britain, West Cornwall, Lizard, Kynance Cove, PWJ, 1986 | BM | 49.9753 | -5.2344 |
| 11 | S. liparina | Great Britain, Kynance, Gew-graze Valley, Kynance Farm, Soap Rock, Gew-graze, PWJ, 1986 | BM | 49.9805 | -5.2273 |
| 12 | S. liparina | Greece, Crete, Kissou Kampos - Aktounta, 540 m, JK & MS, 2017 | SAV0010529 | 35.1872 | 24.5476 |
| 13 | S. liparina | Italy, Toscana, Vescovado di Murlo - Casciano, Poggio Meta, 380 m, LP, 2020 | SAV0017490 | 43.1396 | 11.3301 |
| 14 | S. liparina | Italy, Casciano di Murlo, Casciano di Murlo - Capella Orsa, 460 m, AG & LP, 2013 | SAV0010542, SAV0010549, SAV0010553, SAV0040554, SAV0010559 | 43.1405 | 11.3440 |
| 15 | S. liparina | Italy, Toscana, Vescovado di Murlo, Casciano, Poggio Abbù, 460 m, LP, 2020 | SAV0017475, SAV0017476, SAV0017474, SAV0017484 | 43.1406 | 11.3439 |
| 16 | S. liparina | Italy, Toscana, Vescovado di Murlo, Rocca di Crevole, 290 m, AG & LP, 2013; ibid. ZF & LP, 2018; ibid. LP, 2020 | SAV0017081, SAV0017478, SAV0017479, SAV0017480, SAV0017481, SAV0017482 | 43.1838 | 11.3545 |
| 17 | S. liparina | Italy, Toscana, Pievescola, Gabbra, 250 m, LP, 2013; ibid. ZF, AG & LP, 2013 | SAV0009702, SAV0009703, SAV0009704, SAV0010544, SAV0010545, SAV0010664 | 43.3255 | 11.1279 |
| 18 | S. liparina | Italy, Pievescola, Castello di Casole, 250 m, LP, 2013; ibid. ZF, AG & LP, 2013 | SAV0010558, SAV0010556, SAV0010560, SAV0010561, SAV0010562, SAV0010563, SAV0010565, SAV0010566 | 43.3263 | 11.0942 |
| 19 | S. liparina | Italy, Savona, Varazze, Rianello, CS, 1930 | BP71242 | 44.3632 | 8.5487 |
| 20 | S. liparina | Italy, Liguria, Cerusa, Voltri, FBG | TO | 44.4578 | 8.7867 |
| 21 | S. liparina | Italy, Liguria, Gavi – Bosio, Capanne di Marcarolo, 620 m, AG, MS, JK & KO, 2013 | SAV0010546, SAV0010547 | 44.5556 | 8.7739 |
| 22 | S. liparina | Italy, Lombardia, Pavia, Brallo di Pregola, Il Groppo, 950 m, ZF & LP, 2022 | SAV0017458 | 44.7500 | 9.2720 |
| 23 | S. liparina | Serbia, Kopaonik Mts, Vlajkovci, Brus, 670 m, AG, MS, JK & EŠ, 2016 | SAV0010531 | 43.3390 | 20.9419 |
| 24 | S. liparina | Turkey, Hatay, Gaziantep, Nurdagı, Terken Köyü civarı, 950 m, MCA & JV, 2014 | herb. J. Vondrák 12581 | 37.1036 | 37.0347 |

**Table S2.** Nineteen bioclimatic variables (WorldClim 2 (http://www. worldclim.org/) with information on reduced set of bioclimatic variables after multicollinearity test used for habitat suitability modelling.

| **Climatic variable** | **selection after multicollinearity**  **test** |
| --- | --- |
| b1 = Annual Mean Temperature | no |
| b2 = Mean Diurnal Range (Mean of monthly (max temp - min temp)) | yes |
| b3 = Isothermality (bio2/ bio7) (×100) | no |
| b4 = Temperature Seasonality (standard deviation ×100) | no |
| b5 = Max Temperature of Warmest Month | no |
| b6 = Min Temperature of Coldest Month | no |
| b7 = Temperature Annual Range (bio5- bio6) | yes |
| b8 = Mean Temperature of Wettest Quarter | yes |
| b9 = Mean Temperature of Driest Quarter | yes |
| b10 = Mean Temperature of Warmest Quarter | no |
| b11 = Mean Temperature of Coldest Quarter | no |
| b12 = Annual Precipitation | no |
| b13 = Precipitation of Wettest Month | yes |
| b14 = Precipitation of Driest Month | yes |
| b15 = Precipitation Seasonality (Coefficient of Variation) | yes |
| b16 = Precipitation of Wettest Quarter | no |
| b17 = Precipitation of Driest Quarter | no |
| b18 = Precipitation of Warmest Quarter | yes |
| b19 = Precipitation of Coldest Quarter | yes |

**Table S3.** List of specimens from which genetic data were obtained, with their classification into bioclimatic zones according to Metzger et al. (2013) and into geographical areas (cf. Fačkovcová et al. 2020). Information on locality comprises data as follows: country, the nearest town/village/name of the place, altitude in m a. s. l., collectors' abbreviations (complete list given below), and year. Information on collection includes abbreviation of herbarium according to Index Herbariorum (https://sweetgum.nybg.org/science/ih/) with the code/number of specimens if available. Geographical position (latitude and longitude) is given. Codes of DNA isolates are given as well (column Sample).

Collectors abbreviations: AG – A. Guttová (Bérešová); EŠ - E. Štubňová; ET - E. Timdal; JK - J. Kučera; KO - ; JPH - J. P. Halda; JV - J. Vondrák; LP - L. Paoli; KP - K. Piknová; MCA - M. Candan; MS - M. Slovák; MV - M. Valachovič; MZ - M. Zmrhalová; RT - R. Tsonev; TP - T. Pisani; ZF - Z. Fačkovcová.

| **No** | **Taxon** | **Sample** | **Locality** | **specimen** | **Bioclimatic zone** | **Geography** | **lat** | **long** |
| --- | --- | --- | --- | --- | --- | --- | --- | --- |
| **1** | ***S. liparina*** | **AL_L_1_1** | Albania, Piskupat, 700 m, AG & MS, 2021 | SAV0010600 | K. Warm temperate and mesic | III. Balkan | 41.0205 | 20.6361 |
| **2** | ***S. liparina*** | **AL_L_1_2** | Albania, Piskupat, 700 m, AG & MS, 2021 | SAV0010601 | K. Warm temperate and mesic | III. Balkan | 41.0205 | 20.6361 |
| **3** | ***S. liparina*** | **AL_L_1_3** | Albania, Piskupat, 700 m, AG & MS, 2021 | SAV0010602 | K. Warm temperate and mesic | III. Balkan | 41.0205 | 20.6361 |
| **4** | ***S. liparina*** | **AL_L_1_4** | Albania, Piskupat, 700 m, AG & MS, 2021 | SAV0010608 | K. Warm temperate and mesic | III. Balkan | 41.0205 | 20.6361 |
| **5** | ***S. liparina*** | **AL_L_1_5** | Albania, Piskupat, 700 m, AG & MS, 2021 | SAV0010609 | K. Warm temperate and mesic | III. Balkan | 41.0205 | 20.6361 |
| **6** | ***S. liparina*** | **AL_L_2_1** | Albania, Mollas, 910 m, AG & MS, 2021 | SAV0010610 | J. Cool temperate and moist | III. Balkan | 40.4619 | 20.6675 |
| **7** | ***S. liparina*** | **AL_L_2_2** | Albania, Mollas, 910 m, AG & MS, 2021 | SAV0010611 | J. Cool temperate and moist | III. Balkan | 40.4619 | 20.6675 |
| **8** | ***S. liparina*** | **AL_L_2_3** | Albania, Mollas, 910 m, AG & MS, 2021 | SAV0010612 | J. Cool temperate and moist | III. Balkan | 40.4619 | 20.6675 |
| **9** | ***S. liparina*** | **BiH_L_1_1** | Bosna and Hercegovina, Smolin Mts, Milankovići, Krivaja 450 m, AG & MS, 2021 | SAV0010603 | J. Cool temperate and moist | III. Balkan | 44.2230 | 18.4884 |
| **10** | ***S. liparina*** | **BiH_L_1_2** | Bosna and Hercegovina, Smolin Mts, Milankovići, Krivaja 450 m, AG & MS, 2021 | SAV0010604 | J. Cool temperate and moist | III. Balkan | 44.2230 | 18.4884 |
| **11** | ***S. liparina*** | **BiH_L_1_3** | Bosna and Hercegovina. Smolin Mts. Milankovići. Krivaja 450 m. AG & MS. 2021 | SAV0010605 | J. Cool temperate and moist | III. Balkan | 44.2230 | 18.4884 |
| **12** | ***S. liparina*** | **BiH_L_1_4** | Bosna and Hercegovina, Smolin Mts, Milankovići, Krivaja 450 m, AG & MS, 2021 | SAV0010606 | J. Cool temperate and moist | III. Balkan | 44.2230 | 18.4884 |
| **13** | ***S. liparina*** | **BiH_L_1_5** | Bosna and Hercegovina, Smolin Mts, Milankovići, Krivaja 450 m, AG & MS, 2021 | SAV0010607 | J. Cool temperate and moist | III. Balkan | 44.2230 | 18.4884 |
| **14** | ***S. liparina*** | **CZ_L_1_1** | Czech Republic, Raškov, Vysoký kámen, 530 m, AG, LP, JPH & MZ, 2011 | SAV0010532 | G. Cold and mesic | V. Central Europe | 50.0447 | 16.8877 |
| **15** | ***S. liparina*** | **CZ_L_1_2** | Czech Republic, Raškov, Vysoký kámen, 530 m, AG, LP, JPH & MZ, 2011 | SAV0010532 | G. Cold and mesic | V. Central Europe | 50.0447 | 16.8877 |
| **16** | ***S. liparina*** | **CZ_L_1_3** | Czech Republic, Raškov, Vysoký kámen, 530 m, AG, LP, JPH & MZ, 2011 | SAV0010532 | G. Cold and mesic | V. Central Europe | 50.0447 | 16.8877 |
| **17** | ***S. liparina*** | **FR_L_1_1** | France, Limousine, Haute Vienne, La Roche – l´Abeille, 400 m, AG & LP, 2012 | SAV0010540 | J. Cool temperate and moist | II. Atlantic | 45.6070 | 1.2474 |
| **18** | ***S. liparina*** | **FR_L_1_2** | France, Limousine, Haute Vienne, Magnac Bourg, 440 m, AG & LP, 2012 | SAV0010534 | J. Cool temperate and moist | II. Atlantic | 45.5828 | 1.3955 |
| **19** | ***S. liparina*** | **FR_L_1_3** | France, Limousine, Haute Vienne, Magnac Bourg, 440 m, AG & LP, 2012 | SAV0010537 | J. Cool temperate and moist | II. Atlantic | 45.5828 | 1.3955 |
| **20** | ***S. liparina*** | **FR_L_1_4** | France, Limousine, Haute Vienne, Magnac Bourg, 440 m, AG & LP, 2012 | SAV0010533 | J. Cool temperate and moist | II. Atlantic | 45.5828 | 1.3955 |
| **21** | ***S. liparina*** | **IT_L_1_1** | Italy, Toscana, Vescovado di Murlo, Rocca di Crevole, 320 m, AG & LP, 2013 | SAV0017482 | K. Warm temperate and mesic | I. Apennine | 43.1838 | 11.3545 |
| **22** | ***S. liparina*** | **IT_L_1_4** | Italy. Toscana. Vescovado di Murlo. Casciano, Poggio Meta, 380 m, LP, 2020 | SAV0017491 | K. Warm temperate and mesic | I. Apennine | 43.1396 | 11.3301 |
| **23** | ***S. liparina*** | **IT_L_2_1** | Italy, Toscana, Pievescola, Gabbra, 250 m, ZF, AG & LP, 2013 | SAV0017478 | K. Warm temperate and mesic | I. Apennine | 43.3255 | 11.1279 |
| **24** | ***S. liparina*** | **IT_L_2_2** | Italy, Toscana, Pievescola, Gabbra, 250 m, ZF, AG & LP, 2013 | SAV0017479 | K. Warm temperate and mesic | I. Apennine | 43.3255 | 11.1279 |
| **25** | ***S. liparina*** | **IT_L_2_3** | Italy, Toscana, Pievescola, Gabbra, 250 m, ZF, AG & LP, 2013 | SAV0017480 | K. Warm temperate and mesic | I. Apennine | 43.3255 | 11.1279 |
| **26** | ***S. liparina*** | **IT_L_3_1** | Italy, Liguria, Gavi – Bosio, Capanne di Marcarolo, 620 m, AG, MS, JK & KO, 2013 | SAV0010546 | J. Cool temperate and moist | I. Apennine | 44.5556 | 8.7739 |
| **27** | ***S. liparina*** | **IT_L_3_2** | Italy, Liguria, Gavi – Bosio, Capanne di Marcarolo, 620 m, AG, MS, JK & KO, 2013 | SAV0010547 | J. Cool temperate and moist | I. Apennine | 44.5556 | 8.7739 |
| **28** | ***S. liparina*** | **SRB_L_1_1** | Serbia, Kopaonik Mts, Vlajkovci, Brus, 670 m, AG, MS, JK & EŠ, 2016 | SAV0010531 | H. Cool temperate and dry | III. Balkan | 43.3390 | 20.9419 |
| **29** | ***S. liparina*** | **SRB_L_1_2** | Serbia, Kopaonik Mts, Vlajkovci, Brus, 670 m, AG, MS, JK & EŠ, 2016 | SAV0010531 | H. Cool temperate and dry | III. Balkan | 43.3390 | 20.9419 |
| **30** | ***S. liparina*** | **SRB_L_1_3** | Serbia, Kopaonik Mts, Vlajkovci, Brus, 670 m, AG, MS, JK & EŠ, 2016 | SAV0010531 | H. Cool temperate and dry | III. Balkan | 43.3390 | 20.9419 |
| **31** | ***S. liparina*** | **TR_L_1_2** | Turkey, Hatay, Gaziantep, Nurdagı, Terken Köyü civarı, 950 m, MCA & JV, 2014 | herb. J. Vondrák 12581 | K. Warm temperate and mesic | VI. East Mediterranean | 37.1036 | 37.0347 |
| **32** | ***S. liparina*** | **TR_L_1_3** | Turkey, Hatay, Gaziantep, Nurdagı, Terken Köyü civarı, 950 m, MCA & JV, 2014 | herb. J. Vondrák 12581 | K. Warm temperate and mesic | VI. East Mediterranean | 37.1036 | 37.0347 |
| **33** | ***S. cesatii*** | **IT_CES** | Italy, Trentino - Alto Adige, Malga Zures, 660 m, AG & MS, 2012 | SAV0010170 | K. Warm temperate and mesic | I. Apennine | 43.2924 | 11.1902 |
| **34** | ***S. cesatii*** | **SK_CES** | Slovakia, Veľká Fatra Mts., Ružomberok, Veľká skala, 600 m, AG, 2010 | SAV0010204 | G. Cold and mesic | V. Central Europe | 49.0594 | 19.2787 |
| **35** | ***S. cesatii*** | **BG_CES** | Bulgaria, Vračanski Balkan Mts, Čelopek, Vezhdata, Bazova mogila Mt., 750 m, AG, ZF, MV & RT, 2019 | SAV0010673 | H. Cool temperate and dry | III. Balkan | 43.1372 | 23.6003 |
| **36** | ***S. cesatii*** | **MK_CES** | Northern Macedonia, Drimkolska klisura, Lukovo, 620 m, AG, MS & JK, 2010 | SAV0010964 | J. Cool temperate and moist | III. Balkan | 41.3713 | 20.5968 |
| **37** | ***S. candicans*** | **JO_CAN** | Jordan, Ajloun, 1010 m, MS 2023 | SAV0010626 | L. Warm temperate and xeric | VI. East Mediterranean | 32.3258 | 35.7232 |
| **38** | ***S. candicans*** | **LB_CAN** | Lebanon, Mount Lebanon Governorate, Jour El Bawashek (Jwar El Bawashek), 1070 m, AG, MS & JK, 2019 | SAV0010631 | K. Warm temperate and mesic | VI. East Mediterranean | 33.9945 | 35.6937 |
| **39** | ***S. candicans*** | **IL_CAN** | Israel, Golan, Ein Kinya, limestone outcrops, 680 m, AG, JK & MS, 2014 | SAV0010971 | L. Warm temperate and xeric | VI. East Mediterranean | 33.2288 | 35.7310 |
| **40** | ***S. candicans*** | **SK_CAN** | Slovakia, Malé Karpaty Mts., Sološnica, Malá Vápenná (Malý Roštún), 570 m, KP & AG, 2010 | SAV0009810 | H. Cool temperate and dry | V. Central Europe | 48.5078 | 17.3171 |
| **41** | ***S. candicans*** | **IT_CAN** | Italy, Toscana, Gerfalco, Colline Metallifere Mts., le Cornate, 1030 m, AG, LP & TP, 2010 | SAV0009908 | J. Cool temperate and moist | I. Apennine | 43.1550 | 10.9586 |
| **42** | ***S. candicans*** | **MNE_CAN** | Montenegro, Petrovac - Bukovic, 660 m, ET, 2010 | O-L163454 | K. Warm temperate and mesic | III. Balkan | 42.2202 | 19.0280 |

**References**

Metzger M.J. et al. 2013: High-resolution bioclimate map of the world. Global Ecology and Biogeography 22: 630–638.

**Table S4.** Details of primers, PCR components and cycling parameters.

| **DNA region** | **Description** | **Primers** | **Reference** | **Cycling parameters** |
| --- | --- | --- | --- | --- |
| **ITS** | ITS1 5.8S ITS2 rDNA mycobiont – nuclear, multicopy | ITS 5, ITS 4 | White et al. 1990 | Guttová et al. 2014 |
| **KS** | ketosynthase (KS) domain of the polyketide synthase gene (PKS) | LC1-Im, LC2c-Im | Schmitt et al. 2005 | Guttová et al. 2014 |
| ***Mcm7*** | replication licencing factor | MCM7-solF, MCM7-solR | Guttová et al. 2014 | Guttová et al. 2014 |
| ***rbcl*** | photobiont – chloroplast, | rbcl_203f, rbcl-991r | Nelsen et al. 2011 | initiation: 95 °C , 5 min., 34 cycles: denaturation (95 °C, 45 sek.), annealing (47 °C, 1,30 min.), elongation (72 °C, 2 min.); final elongation 72 °C, 10 min.; 4 °C |
| **ITS** | ITS1 5.8S ITS2 rDNA photobiont – nuclear, multicopy | ITS1T, ITSrPS | Kroken & Taylor 2000 | initiation: 94 °C , 2 min., 10 cycles touch down: 94 °C , 1 min., 62 °C-0,5 °C 1 min., 72 °C 1 min.; 35 cycles: denaturation (94 °C, 1 min.), annealing (57 °C, 1 min.), elongation (72 °C, 1 min.); final elongation 72 °C, 7 min.; 4 °C |

PCR reactions were performed with illustraTM Hot Start Mix RTG PCR beads (GE Healthcare, Buckinghamshire, UK). The PCR products were purified by a mixture of Exonuclease I and FastAP Thermosensitive Alkaline Phosphatase (Thermo Fisher Scientific Inc., Waltham, USA) following the manufacturers’ protocols. GenBank accession numbers are provided in Table S16.

Guttová A, Zozomová-Lihová J, Timdal E, Kučera J, Slovák M, Piknová K, Paoli L. 2014. First insights into genetic diversity and relationships of European taxa of *Solenopsora* (Catillariaceae, Ascomycota) with implications for their delimitation. Bot J Linn Soc 176:203–223

Kroken S & Taylor JW. 2000. Phylogenetic species, reproductive mode, and specificity of the green alga Trebouxia forming lichens with the fungal genus Letharia. Bryologist, 103: 645–660.

Schmitt I, Crespo A, Divakar PK, Fankhauser JD, Herman-Sackett E, Kalb K, Nelsen MP, Rivas-Plata E, Shimp AD,Widhelm T, Lumbsch HT. 2009. Newprimers for promising single-copy genes in fungal phylogenetics and systematics. Persoonia 23: 35–40.

White TJ, Bruns TD, Lee SB, Taylor JW. 1990. Amplification and direct sequencing of fungal ribosomal RNA genes for phylogenetics. In: Innis MA, Gelfand DH, Sninsky JJ, White TJ (eds), PCR protocols: a guide to methods and applications. Academic Press, New York, pp. 315–322

**Table S5.** MaxEnt models variable importance metrics.

|  | Jacknife test – with only variable | Jacknife test – without variable | permutation importance |
| --- | --- | --- | --- |
| *S. liparina* | b19, b13, b14 | b2, b15, b7 | b13, b14, b9 |
| *S. candicans* | b19, b7, b13 | b19, b14, b15/b18 | b2, b9, b13 |
| *S. cesatii* | b18, b13, b14 | b14, b18, b13/b19 | b13, b8, b15 |

**Table S6.** Results of niche similarity and equivalency tests between the *Solenopsora* congeners.

| Comparison | D-value | P-value (Niche Similarity Test) | P-value (Niche Equivalency Test) |
| --- | --- | --- | --- |
| *S. candicans* vs. *S. cesatii* | 0.358 | 0.147 | 0.002 |
| *S. candicans* vs. *S. liparina* | 0.354 | 0.263 | 0.162 |
| *S. cesatii* vs. *S. liparina* | 0.263 | 0.204 | 0.061 |

**Table S7.** Prevalence of Ascomycota and Chlorophyta in metagenomic profiles of *Solenopsora liparina* thalli – detail account. QC – quality control.

| Item | Raw values/ range | Mean ± SD/note | References/note |
| --- | --- | --- | --- |
| Total reads per sample (before QC) | 20,419 – 121,268 | 42,457 ± 27,933 | Table S8 |
| Reads after QC and filtering | – | 24,679 ± 12,778 | Table S8 |
| Reads identified as *S. liparina* mycobiont | – | 18,971 ± 12,660 | Table S8 |
| Non-mycobiont reads per sample | – | 5,615 ± 2,089 | Table S9–S12 |
| Dominant non-mycobiont groups | Chlorophyta, Ascomycota | – | Table S9–S10, Fig. S9 |
| Photobiont OTUs (initial / refined) | 113 → 29 | – | Table S13 |
| Photobiont fraction of non-mycobiont reads | – | 83% ± 19.9% | Table S10, S12 |
| Average photobiont OTUs per sample | ≥3 | 5.9 ± 3.6 | Table S13 |
| Endolichenic fungi fraction | 0.1 – 31.8% | 5.5 ± 8.0% | Table S14 |
| Endolichenic fungi OTUs (initial / refined) | 90 → 54 | – | Table S14 |
| Average OTUs per sample (endolichenic fungi) | – | 6.2 ± 3.9 | 7 samples had ≥10 OTUs, Table S15 |

All *S*. *liparina* samples were sequenced with sufficient depth, ranging from 20,419 to 121,268 reads per sample (average 42,457 ± 27,933). After quality control, filtering, and removal of global OTUs with fewer than five reads, an average of 24,679 ± 12,778 reads per sample remained (Table S8). Following taxonomic assignment, a substantial proportion of reads (average 18,971 ± 12,660) were identified as *S*. *liparina* mycobiont, representing on average 71.7% ± 16% of all reads (Table S8). Distribution of the most abundant 24 OTUs identified as *S. liparina* containing more than 100 sequences are presented in Fig. S16. After removing the primary mycobiont reads and unidentified OTUs, the remaining non-mycobiont reads averaged 5,615 ± 2,089 per sample. In most of the analyzed samples, Chlorophyta (Viridiplantae) was the dominant group, followed by the fungal phylum Ascomycota (Table S9, S10, Fig. S9, S10). Other phyla and higher taxonomic ranks (Anthophyta, Bryophyta, Chytridiomycota, and unclassified reads) contributed only marginally to the non-mycobiont reads and were therefore considered negligible (Table S9–S12). While the proportions of Chlorophyta and Ascomycota varied among samples, significant differences were detected between several populations linked to the samples of *S. liparina* (PERMANOVA, F = 3.11, p = 0.011).

Photobiont reads were initially clustered into 113 OTUs, which were subsequently refined to 29 distinct OTUs (Table S13). In most samples, photobionts represented the dominant non-mycobiont group, accounting for an average of 83% ± 19.9% of non-mycobiont reads (Table S10, S12). Photobiont communities typically comprised at least three or more OTUs per sample (average 5.9 ± 3.6), usually including one dominant OTU along with several minor OTUs of low relative read abundance (Table S13).

Endolichenic fungi constituted only a minor fraction of non-mycobiont reads, ranging from 0.1% to 31.8% (average 5.5 ± 8.0%). Initially, 90 OTUs of endolichenic fungi (represented solely by endolichenic fungi) were identified, which were subsequently clustered into 54 distinct OTUs. The number of OTUs per sample averaged 6.2 ± 3.9, with only seven samples containing 10 or more OTUs (Table S15).

**Table S8.** Overview of metabarcoding dataset description and sequencing results (green cells – *S. liparina*, yellow cells – *S. candicans*, blue cells – *S. cesatii*). The following information is given for each sample: total number of raw reads, number of reads that passed quality filter, number of reads after ITS2 extraction and discarding of global OTUs with less than 5 reads. Within the OTUs, the number of mycobiont reads, their % proportion, and number of non-mycobiont reads are given. In detail, % proportion of 3 the following groups of reads (after discarded mycobiont and unindentified reads) are given – photobiont, endolichenic fungi and others. Abbreviations: qm – quality score minimum, minlen – minimum read length, SD – standard deviation. For details to sample codes see Table S3.

| **No.** | **Sample** | **Raw reads (0 mismatch in tags allowed)** | **Reads after quality filtering (-qm 20, -minlen 40)** | **Reads after ITS2 extraction** | **Reads after discarding global OTUs with less than 5 reads** | **Mycobiont reads** | **Proportion (%) of mycobiont reads** | **Unidentified reads** | **Non-mycobiont reads (after discarding unidentified reads)** | **Proportion (%) of non-mycobiont reads after discarding mycobiont and unidentified reads** | | |
| --- | --- | --- | --- | --- | --- | --- | --- | --- | --- | --- | --- | --- |
|  |  |  |  |  |  |  |  |  |  | **Photobiont** | **Endolichenic funig** | **Others** |
| 1 | AL_L_1_1 | 34986 | 32411 | 26552 | 26234 | 16440 | 62.667 | 8 | 9786 | 98.385 | 1.001 | 0.613 |
| 2 | AL_L_1_2 | 27118 | 25461 | 24166 | 23841 | 17019 | 71.385 | 4 | 6818 | 93.722 | 4.488 | 1.789 |
| 3 | AL_L_1_3 | 32577 | 24200 | 22176 | 22155 | 18319 | 82.686 | 0 | 3836 | 99.114 | 0.547 | 0.339 |
| 4 | AL_L_1_4 | 34853 | 31800 | 29832 | 29739 | 23054 | 77.521 | 16 | 6669 | 99.100 | 0.675 | 0.225 |
| 5 | AL_L_1_5 | 39201 | 37193 | 28974 | 28762 | 20642 | 71.768 | 12 | 8108 | 96.473 | 0.740 | 2.787 |
| 6 | AL_L_2_1 | 29036 | 27376 | 24025 | 23935 | 14875 | 62.147 | 1 | 9059 | 97.870 | 0.706 | 1.424 |
| 7 | AL_L_2_2 | 31720 | 30085 | 24207 | 23900 | 17139 | 71.711 | 15 | 6746 | 92.025 | 5.648 | 2.327 |
| 8 | AL_L_2_3 | 30026 | 27961 | 26067 | 25981 | 16419 | 63.196 | 12 | 9550 | 98.513 | 1.089 | 0.398 |
| 9 | BiH_L_1_1 | 31437 | 29802 | 28481 | 28019 | 21073 | 75.210 | 12 | 6934 | 97.837 | 1.154 | 1.010 |
| 10 | BiH_L_1_2 | 23269 | 21618 | 19296 | 19202 | 12790 | 66.608 | 82 | 6330 | 95.529 | 2.496 | 1.975 |
| 11 | BiH_L_1_3 | 30156 | 28609 | 24464 | 24378 | 15992 | 65.600 | 313 | 8073 | 98.068 | 0.756 | 1.177 |
| 12 | BiH_L_1_4 | 34593 | 32389 | 29250 | 29107 | 21857 | 75.092 | 135 | 7115 | 96.781 | 1.911 | 1.307 |
| 13 | BiH_L_1_5 | 40282 | 37666 | 32342 | 32240 | 21497 | 66.678 | 96 | 10647 | 97.220 | 0.103 | 2.677 |
| 14 | CZ_L_1_1 | 28504 | 27189 | 21340 | 21029 | 14961 | 71.145 | 129 | 5939 | 49.268 | 24.566 | 26.166 |
| 15 | CZ_L_1_2 | 20419 | 19579 | 16429 | 16338 | 9812 | 60.056 | 6 | 6520 | 96.227 | 1.941 | 1.832 |
| 16 | CZ_L_1_3 | 29948 | 29487 | 25985 | 25883 | 18823 | 72.723 | 3 | 7057 | 94.459 | 1.242 | 4.298 |
| 17 | FR_L_1_1 | 27366 | 26729 | 23359 | 23173 | 19002 | 82.001 | 10 | 4161 | 86.037 | 3.965 | 9.998 |
| 18 | FR_L_1_2 | 26421 | 25579 | 21656 | 21370 | 16857 | 78.882 | 3 | 4510 | 61.397 | 15.676 | 22.927 |
| 19 | FR_L_1_3 | 30197 | 29277 | 25384 | 25158 | 21764 | 86.509 | 94 | 3300 | 93.818 | 2.364 | 3.818 |
| 20 | FR_L_1_4 | 20537 | 19740 | 16202 | 15899 | 13153 | 82.728 | 1 | 2745 | 84.699 | 1.494 | 13.807 |
| 21 | IT_L_1_1 | 31832 | 30972 | 27185 | 26854 | 22624 | 84.248 | 128 | 4102 | 63.798 | 6.875 | 29.327 |
| 22 | IT_L_1_4 | 83726 | 47031 | 8058 | 7221 | 4131 | 57.208 | 236 | 2854 | 75.298 | 0.684 | 24.018 |
| 23 | IT_L_2_1 | 30185 | 29642 | 25752 | 25615 | 19774 | 77.197 | 132 | 5709 | 93.659 | 0.753 | 5.588 |
| 24 | IT_L_2_2 | 32921 | 31846 | 27215 | 27142 | 22312 | 82.205 | 12 | 4818 | 97.572 | 0.747 | 1.681 |
| 25 | IT_L_2_3 | 21583 | 21132 | 17327 | 17086 | 12704 | 74.353 | 772 | 3610 | 83.380 | 1.828 | 14.792 |
| 26 | IT_L_3_1 | 33822 | 32776 | 26557 | 26241 | 19761 | 75.306 | 140 | 6340 | 57.303 | 31.782 | 10.915 |
| 27 | IT_L_3_2 | 26979 | 24083 | 21584 | 21430 | 20061 | 93.612 | 150 | 1219 | 86.382 | 6.153 | 7.465 |
| 28 | SRB_L_1_1 | 30527 | 29393 | 23540 | 22979 | 17690 | 76.983 | 20 | 5269 | 93.813 | 2.202 | 3.986 |
| 29 | SRB_L_1_2 | 26804 | 23313 | 19951 | 19863 | 16435 | 82.742 | 3 | 3425 | 92.000 | 2.978 | 5.022 |
| 30 | SRB_L_1_3 | 31344 | 30747 | 26260 | 26042 | 21429 | 82.286 | 7 | 4606 | 97.894 | 0.847 | 1.259 |
| 31 | TR_L_1_2 | 29243 | 28713 | 25842 | 25437 | 19542 | 76.825 | 15 | 5880 | 81.241 | 1.854 | 16.905 |
| 32 | TR_L_1_3 | 27360 | 24864 | 21029 | 20921 | 15152 | 72.425 | 7 | 5762 | 34.658 | 2.499 | 62.843 |
| 33 | IL_CAN | 101301 | 53876 | 11027 | 9406 | 2415 | 25.675 | 217 | 6774 | 88.810 | 1.550 | 9.640 |
| 34 | IT_CAN | 74483 | 40803 | 7799 | 6649 | 1560 | 23.462 | 148 | 4941 | 78.527 | 1.538 | 19.935 |
| 35 | JO_CAN | 20859 | 19676 | 15755 | 15380 | 10117 | 65.780 | 94 | 5169 | 84.252 | 2.863 | 12.885 |
| 36 | LB_CAN | 22549 | 22074 | 18581 | 18390 | 15707 | 85.411 | 6 | 2677 | 81.696 | 14.195 | 4.109 |
| 37 | MNE_CAN | 80244 | 44078 | 5877 | 5008 | 1954 | 39.018 | 150 | 2904 | 93.079 | 1.274 | 5.647 |
| 38 | SK_CAN | 110150 | 60352 | 10253 | 8976 | 3289 | 36.642 | 69 | 5618 | 55.376 | 24.973 | 19.651 |
| 39 | BG_CES | 33344 | 31965 | 27963 | 27775 | 21155 | 76.166 | 46 | 6574 | 66.322 | 14.664 | 19.014 |
| 40 | IT_CES | 106010 | 80171 | 66063 | 63760 | 57672 | 90.452 | 159 | 5929 | 21.572 | 8.922 | 69.506 |
| 41 | MK_CES | 104022 | 69143 | 57819 | 55826 | 53172 | 95.246 | 212 | 2442 | 86.405 | 1.515 | 12.080 |
| 42 | SK_CES | 121268 | 89388 | 74672 | 72175 | 66656 | 92.353 | 203 | 5316 | 30.211 | 28.047 | 41.742 |
|  |  |  |  |  |  |  |  |  |  |  |  |  |
|  | **Average** | 42457,190 | 34052,119 | 25149,905 | 24679,024 | 18971,429 | 71,712 | 92,333 | 5615,262 | 82,614 | 5,507 | 11,879 |
|  | **SD** | 27933,859 | 15330,674 | 13097,066 | 12778,404 | 12660,828 | 16,008 | 133,437 | 2089,453 | 19,870 | 8,047 | 15,399 |
|  | **Median** | 30935,500 | 29564,500 | 24186,500 | 23917,500 | 17414,500 | 75,151 | 33,000 | 5735,500 | 92,012 | 1,883 | 5,305 |
|  | **Minimum** | 20419,000 | 19579,000 | 5877,000 | 5008,000 | 1560,000 | 23,462 | 0,000 | 1219,000 | 21,572 | 0,103 | 0,225 |
|  | **Maximum** | 121268,000 | 89388,000 | 74672,000 | 72175,000 | 66656,000 | 95,246 | 772,000 | 10647,000 | 99,114 | 31,782 | 69,506 |

**Table S9.** Taxonomic identification to the phylum level per populations. Values represent averages (avg). Green cells – *S. liparina*, yellow cells – *S. candicans*, blue cells – *S. cesatii*.

| **Phylum** | **S. liparina - avg. per population** | | | | | | | | | | **Solenopsora - avg. of all populations per species** | | |
| --- | --- | --- | --- | --- | --- | --- | --- | --- | --- | --- | --- | --- | --- |
|  | **A_L_1** | **A_L_2** | **B_L_1** | **CZ_L_1** | **FR_L_1** | **IT_L_1** | **IT_L_2** | **IT_L_3** | **S_L_1** | **TR_L_1** | **liparina** | **candicans** | **cesatii** |
| Anthophyta | 0 | 0.012437 | 0 | 0 | 0 | 0 | 0 | 0 | 0 | 0 | 0.001166 | 0.00023 | 0.000823 |
| Ascomycota | 0.024675 | 0.037691 | 0.02359 | 0.191535 | 0.175374 | 0.174071 | 0.077505 | 0.278214 | 0.049736 | 0.158646 | 0.101065 | 0.182307 | 0.472209 |
| Basidiomycota | 0.001677 | 0.00095 | 0.005509 | 0.000921 | 0.009687 | 0.02249 | 0.007035 | 0.001309 | 0.004574 | 0.261856 | 0.021451 | 0.014385 | 0.015385 |
| Bryophyta | 0 | 0 | 5.77E-05 | 0 | 0 | 0.000244 | 0 | 0 | 0 | 0 | 2.43E-05 | 0 | 0.000266 |
| Ciliophora | 0 | 0 | 0 | 0 | 0 | 0 | 0 | 0 | 0 | 0 | 0 | 0.000178 | 0 |
| Chlorophyta | 0.973589 | 0.948921 | 0.970812 | 0.799846 | 0.814879 | 0.803073 | 0.915367 | 0.718426 | 0.94569 | 0.579498 | 0.875406 | 0.802669 | 0.510183 |
| Chytridiomycota | 0 | 0 | 0 | 0 | 0 | 0 | 0 | 0 | 0 | 0 | 0 | 0 | 0 |
| unclassified | 5.87E-05 | 0 | 3.16E-05 | 0.007698 | 6.01E-05 | 0.000122 | 9.23E-05 | 0.002051 | 0 | 0 | 0.000888 | 0.000232 | 0.001133 |
