# Supplementary material for: Ultramafic specialist lichens persist through flexible symbioses structured by climate-driven filtering and modulated by substrate effects
Source: IMA Fungus. 2026 Aug 24;17:e199447. doi: 10.3897/imafungus.17.199447 (PMC13531026; doi:10.3897/imafungus.17.199447)
Supplement: Supplementary file 3 [file imafungus-17-e199447-s005.docx]

**Ultramafic specialist lichens persist through flexible symbioses structured by climate-driven filtering and modulated by substrate effects**

**Running head: Ultramafic symbiosis dynamics**

**Miroslav Caboň, Marek Slovák, Marek Svitok, Jana Steinová, Zuzana Fačkovcová**, **Jaroslava Gužiková, Jaromír Kučera, Luca Paoli,** **Tomáš Větrovský, Alica Hindáková, Dušan Senko, Judita Zozomová-Lihová, Andrea Melichárková, Zuzana Gajdošová, Anna Guttová**

**Supporting information – figure captions S1–S20**

**Figure S1.** Analytical workflow.

**Fig. S2.** Maximum likelihood phylogeny of studied *Solenopsora* species inferred from concatenated sequences (ITS, KS and *mcm7*) using RAxML under GTR+G model. Node support values are shown as ML bootstrap percentages based on 1000 replicates / Bayesian posterior probabilities. The scale bar represents substitutions per sites. For sample codes of sequences generated for this study see Table S3, other sample codes follow Guttová et al. 2014.

**Fig. S3.** Projection of recent HSM of *Solenopsora candicans* across its range. Colours indicate the predicted habitat suitability ranging from 0.25 (low suitability) to 1.0 (high suitability). Black dots represent known occurrence records of *S. candicans*. Light green shading indicates predicted habitat suitability values < 0.25, which fall below the range represented by the colour scale in the figure legend.

**Fig. S4** Projection of recent HSM of *Solenopsora cesatii* across its range. Colours indicate the predicted habitat suitability ranging from 0.25 (low suitability) to 1.0 (high suitability). Black dots represent known occurrence records of *S. cesatii*.

**Fig. S5.** Predictive estimate of importance of variables used for MaxEnt models – results of Jacknife test for training data of *Solenopsora liparina*. b2 = Mean Diurnal Range, b7 = Temperature Annual Range, b8 = Mean Temperature of Wettest Quarter, b9 = Mean Temperature of Driest Quarter, b13 = Precipitation of Wettest Month, b14 = Precipitation of Driest Month, b15 = Precipitation Seasonality, b18 = Precipitation of Warmest Quarter, b19 = Precipitation of Coldest Quarter.

**Fig. S6.** Predictive estimate of importance of variables used for MaxEnt models – results of Jacknife test for training data of *Solenopsora candicans*. b2 = Mean Diurnal Range, b7 = Temperature Annual Range, b8 = Mean Temperature of Wettest Quarter, b9 = Mean Temperature of Driest Quarter, b13 = Precipitation of Wettest Month, b14 = Precipitation of Driest Month, b15 = Precipitation Seasonality, b18 = Precipitation of Warmest Quarter, b19 = Precipitation of Coldest Quarter.

**Fig. S7.** Predictive estimate of importance of variables used for MaxEnt models – results of Jacknife test for training data of *Solenopsora cesatii*. b2 = Mean Diurnal Range, b7 = Temperature Annual Range, b8 = Mean Temperature of Wettest Quarter, b9 = Mean Temperature of Driest Quarter, b13 = Precipitation of Wettest Month, b14 = Precipitation of Driest Month, b15 = Precipitation Seasonality, b18 = Precipitation of Warmest Quarter, b19 = Precipitation of Coldest Quarter.

**Fig. S8.** Response curves demonstrating how only the corresponding variable contributes to the HSM of *S. liparina*. b2 = Mean Diurnal Range, b7 = Temperature Annual Range, b8 = Mean Temperature of Wettest Quarter, b9 = Mean Temperature of Driest Quarter, b13 = Precipitation of Wettest Month, b14 = Precipitation of Driest Month, b15 = Precipitation Seasonality, b18 = Precipitation of Warmest Quarter, b19 = Precipitation of Coldest Quarter.

**Fig. S9.** Response curves demonstrating how only the corresponding variable contributes to the HSM of *S. candicans*. b2 = Mean Diurnal Range, b7 = Temperature Annual Range, b8 = Mean Temperature of Wettest Quarter, b9 = Mean Temperature of Driest Quarter, b13 = Precipitation of Wettest Month, b14 = Precipitation of Driest Month, b15 = Precipitation Seasonality, b18 = Precipitation of Warmest Quarter, b19 = Precipitation of Coldest Quarter.

**Fig. S10.** Response curves demonstrating how only the corresponding variable contributes to the HSM of *S. cesatii*. b2 = Mean Diurnal Range, b7 = Temperature Annual Range, b8 = Mean Temperature of Wettest Quarter, b9 = Mean Temperature of Driest Quarter, b13 = Precipitation of Wettest Month, b14 = Precipitation of Driest Month, b15 = Precipitation Seasonality, b18 = Precipitation of Warmest Quarter, b19 = Precipitation of Coldest Quarter.

**Fig. S11.** Taxonomic identification of OTUs to the phylum level per samples after removing the primary mycobiont reads. Values represent relative read abundance based on amplicone sequencing data for each sample. For sample codes see Table S3.

**Fig. S12.** Functional guilds per samples after removing the primary mycobiont reads. Values are based on amplicon sequencing data. For sample codes see Table S3. EcM – ectomycorrhizal fungi, ND – not determined.

**Fig. S13.** Diversity metrics of photobiont communities across populations of S. liparina and number of recognized photobiont OTUs. Boxplots show the distribution of six diversity indices: Shannon Index, Inverse Simpson Index, Simpson 1D Index, Dominance Index, and Evenness Index, for each sampled population. Letters above the boxes indicate groupings based on Tukey’s HSD post-hoc test, with shared letters denoting no significant difference among populations within each metric. Details on sample codes are given in Table S3.

**Fig. S14.** Relative abundance reads of endolichenic photobionts. For sample codes see Table S3. Bubble colour corresponds to the studied *Solenopsora* species as follows: LIP – *S. liparina*, CES – *S. cesatii*, CAN – *S. candicans*.

**Figure S15.** Significant relationship between the temperature annual range (b7) and species richness of *S*. *liparina* photobionts. Predictions from a Poisson GLMM (solid line) are shown with 95% confidence intervals (shaded band).

**Fig. S16.** RAxML maximum likelihood phylogeny of studied *Solenopsora* species major photobiont ITS Sanger sequences. Significant bootstrap values are shown at nodes. Accessions from ultramafic specialist *S. liparina* are highlighted in green, accessions from *Solenopsora* congeners growing on calcareous substrates are highlighted in yellow. Reference sequences were retrieved from GenBank based on alignment published by Gheza *et al.* (2025). Details on sample codes are given in Table S3.

**Fig. S17.** RAxML maximum likelihood phylogeny of studied *Solenopsora* species major photobiont *rbcl* Sanger sequences. Significant bootstrap values are shown at nodes. Accessions from ultramafic specialist *S. liparina* are highlighted in green, accessions from *Solenopsora* congeners growing on calcareous substrates are highlighted in yellow. Reference sequences were retrieved from GenBank based on alignment published by Gheza *et al.* (2025). Details on sample codes are given in Table S3.

**Fig. S18.** RAxML maximum likelihood phylogeny of studied *Solenopsora* species photobiont based on amplicons and ITS2 Sanger sequences. Significant bootstrap values are shown at nodes. Accessions from ultramafic specialist *S. liparina* are highlighted in green, accessions from *Solenopsora* congeners growing on calcareous substrates are highlighted in yellow. Reference sequences were retrieved from GenBank based on alignment published by Gheza *et al.* (2025). Details on sample codes are given in Table S3.

**Fig. S19.** Diversity metrics of endolichenic fungi across populations of S. liparina and number of recognized endolichenic fungi OTUs. Boxplots show the distribution of six diversity indices: Shannon Index, Inverse Simpson Index, Simpson 1D Index, Dominance Index, and Evenness Index, for each sampled population. Letters above the boxes indicate groupings based on Tukey’s HSD post-hoc test, with shared letters denoting no significant difference among populations within each metric. Details on sample codes are given in Table S3.

**Fig. S20.** Relative abundance reads of endolichenic fungi. For sample codes see Table S3. Bubble colour corresponds to the studied *Solenopsora* species as follows: LIP – *S. liparina*, CES – *S. cesatii*, CAN – *S. candicans*.

**Supporting information – table captions S1–S17**

**Table S1.** List of occurrence data for *Solenopsora liparina* based on revised specimens and own field study. Information on locality comprises data as follows: country, the nearest town/village/name of the place, altitude in m a. s. l., collectors' abbreviations (complete list given in the caption of Table S 1), and year. Information on collection includes abbreviation of herbarium according to Index Herbariorum (https://sweetgum.nybg.org/science/ih/) with the code/number of specimens, if available. Geographical position (latitude and longitude) is given.

Collectors abbreviations: AG – A. Guttová (Bérešová); CS – C. Sbarbaro; EŠ - E. Štubňová; ET - E. Timdal; FBG – F. Baglietto; JK - J. Kučera; KO - ; JPH - J. P. Halda; JV - J. Vondrák; ; LL – L. Lőkös; LP - L. Paoli; KP - K. Piknová; MCA - M. Candan; MS - M. Slovák; MV - M. Valachovič; MZ - M. Zmrhalová; KO – K. Olšavská; PWJ – P. W. James; RT - R. Tsonev; TP - T. Pisani; ZF - Z. Fačkovcová.

**Table S2.** Nineteen bioclimatic variables (WorldClim 2 (http://www. worldclim.org/) with information on reduced set of bioclimatic variables after multicollinearity test used for habitat suitability modelling.

**Table S3.** List of specimens from which genetic data were obtained, with their classification into bioclimatic zones according to Metzger et al. (2013) and into geographical areas (cf. Fačkovcová et al. 2020). Information on locality comprises data as follows: country, the nearest town/village/name of the place, altitude in m a. s. l., collectors' abbreviations (complete list given below), and year. Information on collection includes abbreviation of herbarium according to Index Herbariorum (https://sweetgum.nybg.org/science/ih/) with the code/number of specimens if available. Geographical position (latitude and longitude) is given. Codes of DNA isolates are given as well (column Sample).

Collectors abbreviations: AG – A. Guttová (Bérešová); EŠ - E. Štubňová; ET - E. Timdal; JK - J. Kučera; KO - ; JPH - J. P. Halda; JV - J. Vondrák; LP - L. Paoli; KP - K. Piknová; MCA - M. Candan; MS - M. Slovák; MV - M. Valachovič; MZ - M. Zmrhalová; RT - R. Tsonev; TP - T. Pisani; ZF - Z. Fačkovcová.

**Table S4.** Details of primers, PCR components and cycling parameters.

**Table S5.** MaxEnt models variable importance metrics.

**Table S6.** Results of niche similarity and equivalency tests between the *Solenopsora* congeners.

**Table S7.** Prevalence of Ascomycota and Chlorophyta in metagenomic profiles of *Solenopsora liparina* thalli – detail account. QC – quality control.

**Table S8.** Overview of metabarcoding dataset description and sequencing results (green cells – *S. liparina*, yellow cells – *S. candicans*, blue cells – *S. cesatii*). The following information is given for each sample: total number of raw reads, number of reads that passed quality filter, number of reads after ITS2 extraction and discarding of global OTUs with less than 5 reads. Within the OTUs, the number of mycobiont reads, their % proportion, and number of non-mycobiont reads are given. In detail, % proportion of 3 the following groups of reads (after discarded mycobiont and unindentified reads) are given – photobiont, endolichenic fungi and others. Abbreviations: qm – quality score minimum, minlen – minimum read length, SD – standard deviation. For details to sample codes see Table S3.

**Table S9.** Taxonomic identification to the phylum level per populations. Values represent averages (avg). Green cells – *S. liparina*, yellow cells – *S. candicans*, blue cells – *S. cesatii*.

**Table S10.** Taxonomic identification to the phylum level per samples. Values represent Relative Reads Abundance for each sample. Green cells – *S. liparina*, yellow cells – *S. candicans*, blue cells – *S. cesatii*.

**Table S11.** Functional guilds per populations. Last three columns represent average (avg.) Relative Reads Abundance values for species. Green cells – *S. liparina*, yellow cells – *S. candicans*, blue cells – *S. cesatii*.

**Table S12.** Functional guilds per samples. Green cells – *S. liparina*, yellow cells – *S. candicans*, blue cells – *S. cesatii*.

**Table S13.** Taxonomic identification of photobiont OTUs based on metabarcoding data. The table lists OTU identifiers, reference sequences, taxonomic classification, sequence similarity and coverage, and relative read abundance across samples after removal of primary mycobiont reads. Sample codes are given in Table S3. **OTUs identity exclusive to** S. liparina **are highlighted in green, and OTUs exclusive to calcareous congeners (**S. candicans **and** S. cesatii**) in blue.** Green cells – *S. liparina*, yellow cells – *S. candicans*, blue cells – *S. cesatii*.

**Table S14.** Taxonomic identification of endolichenic fungal OTUs based on metabarcoding data. The table lists OTU identifiers, reference sequences, taxonomic classification, sequence similarity and coverage, and relative read abundance across samples after removal of primary mycobiont reads. Sample codes are given in Table S3. **OTUs identity exclusive to** S. liparina **are highlighted in green, and OTUs exclusive to calcareous congeners (**S. candicans **and** S. cesatii**) in blue.** Green cells – *S. liparina*, yellow cells – *S. candicans*, blue cells – *S. cesatii*.

**Table S15.** List of Sanger sequences. Details on samples are given in Table S3. Newly generated sequences are marked in bold. Sanger sequencing was carried out at the commercial company Eurofins Genomics, Constance, Germany.

**Table S16.** Summary of explorative analysis testing the effect of environmental predictors on community characteristics of photobionts and endolichenic fungi associated with *S. liparina*. Community composition refers to multivariate variation in species abundance data and was assessed using PERMANOVA based on Bray-Curtis dissimilarities. Table reports test statistics (χ2 for alpha diversity measures, F for evenness, and pseudo-F for community composition) and associated P-values (after the slash). Results significant at α = 0.05 are shown in bold. Note that dashes indicate predictors excluded from the analysis due to collinearity (VIF > 5). b7 = Temperature Annual Range (bio5- bio6), b13 = Precipitation of Wettest Month, b15 = Precipitation Seasonality, b18 = Precipitation of Warmest Quarter, b19 = Precipitation of Coldest Quarter

**Table S17.** Summary of alpha diversity metrics – values of selected diversity indices per sample. Green cells – *S. liparina*, yellow cells – *S. candicans*, blue cells – *S.*
